# Lymphocyte subset-based non-invasive biomarker predicts immunochemotherapy efficacy in EGFR-TKI-pretreated EGFR-mutated NSCLC

**DOI:** 10.1016/j.isci.2025.113211

**Published:** 2025-07-25

**Authors:** Lianxi Song, Liang Zeng, Qinqin Xu, Yizhi Li, Wenhuan Guo, Shaoding Lin, Wenjuan Jiang, Zhan Wang, Li Deng, Zhe Huang, Haoyue Qin, Huan Yan, Xing Zhang, Fan Tong, Ruiguang Zhang, Zhaoyi Liu, Lin Zhang, Juan Yu, Xue Yang, Yang Xia, Xiaorong Dong, Gao Zhang, Nong Yang, Yongchang Zhang

**Affiliations:** 1 Department of Medical Oncology, Lung Cancer and Gastrointestinal Unit, Hunan Cancer Hospital/The Affiliated Cancer Hospital of Xiangya School of Medicine, Central South University, Changsha 410013, China; 2 Graduate Collaborative Training Base of Hunan Cancer Hospital, Hengyang Medical School, University of South China, Hengyang, Hunan 421001, China; 3 Department of Medical Oncology, Qinghai Provincial People’s Hospital, Xining 810000, China; 4 Department of Pathology, Ninth People’s Hospital, Shanghai Jiao Tong University School of Medicine, Shanghai 20025, China; 5 Department of Medical Oncology, The First Affiliated Hospital of Hunan University of Medicine, Huaihua 418000, China; 6 Cancer Center, Union Hospital, Tongji Medical College, Huazhong University of Science and Technology, Wuhan 430022, China; 7 Department of Medical Oncology, the Affiliated Cancer Hospital of Xiangya School of Medicine, Central South University/Hunan Cancer Hospital, Changsha, Hunan 410008, China; 8 Department of Radiotherapy, the Affiliated Cancer Hospital of Xiangya School of Medicine, Central South University/Hunan Cancer Hospital, Changsha, Hunan 410008, China; 9 Department of Medical Oncology, Zhangjiajie People’s Hospital, Zhangjiajie, Hunan 410008, China; 10 Key Laboratory of Carcinogenesis and Translational Research (Ministry of Education), Department of Thoracic Medical Oncology, Peking University Cancer Hospital and Institute, Beijing 100142, China; 11 Department of Respiratory and Critical Care Medicine, Second Affiliated Hospital, Zhejiang University School of Medicine, Hangzhou, Zhejiang 310009, China; 12 Faculty of Dentistry, The University of Hong Kong, 34 Hospital Road, Sai Ying Pun, Hong Kong SAR 999077, China; 13 Department of Medical Oncology, Yiyang Center Hospital, Yiyang 410000, China

**Keywords:** Health sciences, Medicine, Medical specialty, Internal medicine, Oncology

## Abstract

Immune checkpoint inhibitors (ICIs) combined with chemotherapy (Chemo+ICI) have shown variable benefit in patients with epidermal growth factor receptor (*EGFR*)-mutant non-small cell lung cancer (NSCLC) whose disease progressed following tyrosine kinase inhibitors (TKIs) therapy. This retrospective study evaluated treatment outcomes between Chemo+ICI and chemotherapy alone, and developed a lymphocyte subset model (LSM) using pre-treatment blood samples to predict survival outcome. Patients receiving Chemo+ICI showed significantly improved overall response rates (34.2% vs. 22.0%, *p* = 0.04), progression-free survival (6.0 vs. 4.0 months, *p* < 0.0001), and overall survival (14.6 vs. 11.0 months, *p* < 0.001). LSM yielded an area under the curve of 0.726, with 58.6% sensitivity and 83.8% specificity. Across training and validation cohorts, patients classified as LSM-high had significantly longer progression-free survival than those in the LSM-low group. These findings provide real-world clinical evidence supporting the benefit of Chemo+ICI in EGFR-TKI-resistant *EGFR*-mutant NSCLC, and suggest that LSM may help identify patients most likely to benefit from Chemo+ICI.

## Highlights

• Chemo+ICI associated with improved PFS outcomes (6.0 vs. 4.0 months, *p* < 0.001)
• LSM is a non-invasive biomarker for selecting patients who benefit from Chemo+ICI
• LSM (AUC = 0.73) outperforms PD-L1 (AUC = 0.58) and TMB (AUC = 0.63) in PFS prediction

## Graphical abstract

**Figure:**
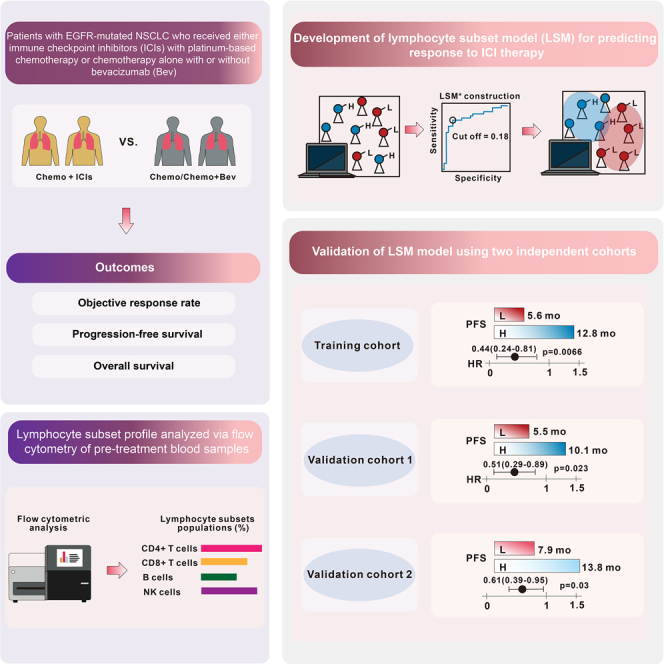

## Introduction

Approximately 50% of East Asian patients diagnosed with advanced non-small cell lung cancer (NSCLC) harbor actionable mutations in epidermal growth factor receptor (*EGFR*).^1^^,^^2^ A large proportion of these patients clinically benefit from molecularly targeted treatment with EGFR tyrosine kinase inhibitors (TKIs) than with chemotherapy.^3^^,^^4^^,^^5^ However, disease progression remains a clinical challenge due to acquisition of resistance from EGFR-TKI, including pathway reactivation with EGFR secondary mutations, pathway bypass through activation of other parallel pathway/s, and pathway indifference such as histological transformation and other unknown resistance mechanisms.^6^ Approximately 20% of patients whose tumors either exhibit histological transformation or do not harbor clinically actionable mutations after developing resistance from EGFR-TKI.^7^ Until now, chemotherapy remains as the standard treatment option for the subset of patients with *EGFR*-mutant NSCLC whose disease progressed from EGFR-TKI therapy without clinically actionable mutations.

Similar to EGFR-TKIs, the efficacy of monoclonal antibodies targeting either programmed death receptor-1 (PD-1) or programmed death receptor ligand-1 (PD-L1) was remarkable for various solid tumors, including NSCLCs that lack actionable mutations.^8^^,^^9^^,^^10^ These immune checkpoint inhibitors (ICIs) combined with the standard platinum-based doublet chemotherapy regimen (Chemo+ICI) are now the recommended first-line treatment option for patients with NSCLC whose tumors do not harbor actionable mutations.^8^^,^^9^^,^^10^ PD-L1 expression and tumor mutation burden (TMB) are considered as biomarkers of ICI response.^11^ A series of preclinical and retrospective studies suggested that activating *EGFR* mutations upregulate PD-L1 expression in NSCLC^12^^,^^13^^,^^14^^,^^15^; however, there is uncertainty as to whether anti-PD-1/anti-PD-L1 inhibitors lead to survival benefit for patients who were previously treated with EGFR-TKIs. Several clinical trials performed subgroup analyses in an attempt to address this important clinical question. The final results from the Checkmate 722 and KEYNOTE-789 failed to show the survival benefits afforded by ICI-based regimens in this patient population; however, encouraging results were demonstrated by an exploratory subgroup analysis of the IMpower150 and ORIENT-31 studies.^16^^,^^17^^,^^18^^,^^19^ Hence, the benefit of ICI-containing regimen in this patient population remains a controversy and warrants an investigation to determine if this regimen can be an optimal subsequent-line treatment strategy. Inconsistent results from these studies may be due to the lack of effective biomarkers for patient selection. This raises an urgent need to identify biomarkers that could effectively identify the subset of patients who would derive benefit from the Chemo+ICI combination therapy upon disease progression on EGFR-TKIs.

Numerous biomarkers, including PD-L1 expression level, TMB, and tumor-infiltrating immune cells (e.g., T and B cell subgroups), are being tested in various clinical practice settings as predictors of ICI response^20^^,^^21^^,^^22^^,^^23^; however, their predictive performances were mainly demonstrated in the first-line setting and suffered when used alone. In order to further improve the predictive ability of these biomarkers, statistical models that combine two or more immunological biomarkers or molecular expression patterns have shown modest enhancement in their performance in predicting clinical outcomes with ICI treatment rather than using just a single biomarker.^24^^,^^25^^,^^26^ For example, Gu et al. reported the potential utility of a LASSO-Cox model based on immune-related gene expression patterns in predicting ICI outcomes.^27^ By integrating genomic, molecular, demographic and clinical data from a cohort composed of 1,479 patients with NSCLC treated with ICI, Chowell et al. developed a machine-learning model to predict ICI response.^26^ These statistical models achieved high sensitivity and specificity in predicting clinical response to ICIs, demonstrating the advantage of applying statistical models that leverage the combined use of multiple biomarkers for predicting clinical benefit.^24^^,^^25^^,^^26^

In this study, we retrospectively analyzed clinical outcomes of patients with NSCLC whose tumors lacked clinically actionable oncogenic mutations after progressing from EGFR-TKIs and who subsequently received either Chemo+ICIs or chemotherapy with or without bevacizumab (Bev). We further explored the clinical utility of a logistic regression-based predictive model to identify potential blood-based immune biomarkers to select the subset of patients with *EGFR*-mutated, EGFR-TKI-resistant NSCLC who will most likely benefit from the combination of Chemo+ICIs.

## Results

### Baseline characteristics of the study cohort

**Figure 1:**
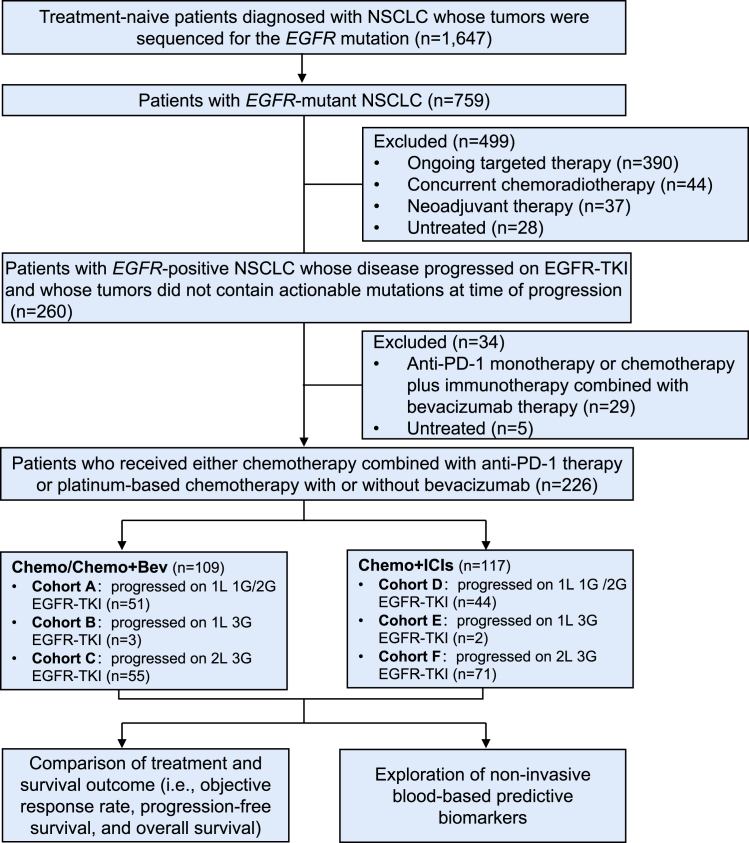
Patient selection Flow diagram illustrates the selection criteria for patients who progressed on prior EGFR-TKI therapy. Abbreviations are as follows: 1G/2G, first-generation/second-generation; 1L, first-line; 2L, second-line; 3G, third-generation.

**Figure 2:**
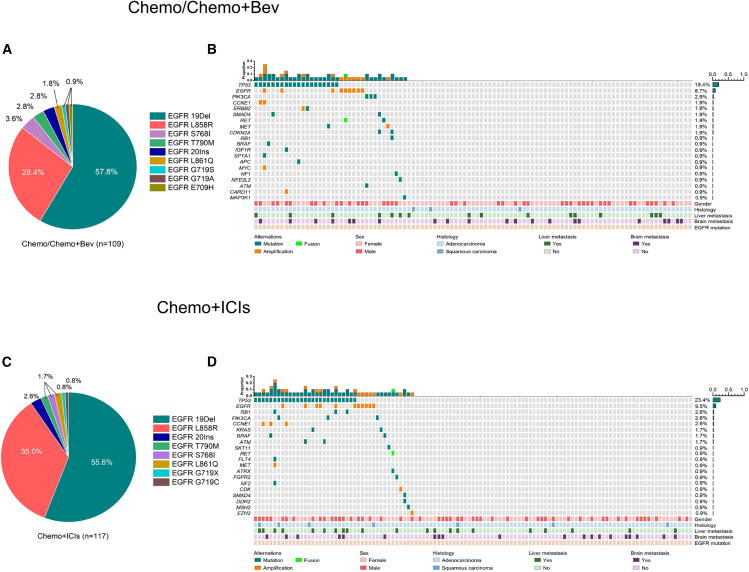
*EGFR* mutational subtypes and mutational landscape (A and B) Distribution of *EGFR* mutation subtypes in patients who received either chemotherapy with/without bevacizumab (Chemo/Chemo+Bev; *n* = 109) (A) or chemotherapy plus immune checkpoint inhibitors (ICIs) (Chemo+ICI; *n* = 117) (B) after EGFR-TKI progression. Mutation types were represented by different colors. (C and D) OncoMap depicting the mutational landscape of patients included in the study before receiving either chemotherapy with/without bevacizumab (Chemo/Chemo+Bev; *n* = 109) (C) or chemotherapy plus immune checkpoint inhibitors (ICIs) (Chemo+ICI; *n* = 117) (D) baseline EGFR-TKI treatment. The clinical details of each patient were annotated at the bottom of each oncomap.

The flow diagram of the patient screening and patient stratification is shown in Figure 1. Table 1 lists the baseline characteristics of enrolled patients. Baseline characteristics showed no difference between these two groups with respect to age, sex, smoking history, Eastern Cooperative Oncology Group performance score (ECOG PS), pathological classification, presence or absence of brain or liver metastasis and relevant gene alterations. Of 226 patients with progressive disease on EGFR-TKI, 109 received Chemo/Chemo+Bev and 117 patients received Chemo+ICIs. *EGFR* mutation status was determined by next-generation sequencing (NGS) in 218 patients (Chemo/Chemo+Bev group, *n* = 103; Chemo+ICIs group, *n* = 115) or amplification refractory mutation system-polymerase chain reaction (ARMS-PCR) in the remaining eight patients. Figure 2 shows the distribution of baseline *EGFR* mutation status for patients who received Chemo/Chemo+Bev or Chemo+ICIs. Specifically, *EGFR* small insertion-deletion in exon 19 (19Del) accounts for the majority of *EGFR* mutations detected in patients who received Chemo/Chemo+Bev (57.8%; 63/109; Figures 2A and 2B) or Chemo+ICIs (55.6%, 65/117; Figures 2C and 2D). In the Chemo+ICIs group, the third-generation EGFR-TKIs (62.4%; 73/117) account for a majority of EGFR-TKIs received prior to receiving ICI-containing regimen. The history of EGFR-TKIs were comparable between the Chemo/Chemo+Bev group and the Chemo+ICIs group (Figure 1).

**Table 1:** Baseline characteristics of the cohort

Clinicopathologic characteristics	Total (*n* = 226)	Chemo+ICI					Chemo/Chemo+Bev					*p*
		Total (*n* = 117)	Cohort A (*n* = 44)	Cohort B (*n* = 2)	Cohort C (*n* = 71)	*p*^a^	Total (*n* = 109)	Cohort D (*n* = 51)	Cohort E (*n* = 3)	Cohort F (*n* = 55)	*p*^b^	
Age—yr. (median, range)	54.7 (27–82)	55.0 (27–82)	55.7 (27–82)	65.5 (65–66)	54.5 (27–74)	0.91	54.0 (31–77)	56.0 (37–74)	56.7 (42–77)	53.8 (31–76)	0.83	0.92
Sex—no. patients (%)	–	–	–	–	–	0.56	–	–	–	–	0.81	0.95
Female	124 (54.9%)	64 (54.8%)	23 (52.3%)	0	41 (57.7%)	–	60 (55.1%)	29 (56.9%)	1 (33.3%)	30 (54.5%)	–	–
Male	102 (45.1%)	53 (45.2%)	21 (47.7%)	2 (100%)	30 (42.3%)	–	49 (44.9%)	22 (43.1%)	2 (66.7%)	25 (45.5%)	–	–
Smoking history—no. patients (%)	–	–	–	–	–	0.75	–	–	–	–	0.93	0.48
Former	84 (37.2%)	46 (39.3%)	18 (40.9%)	1 (50.0%)	27 (38.0%)	–	38 (34.9%)	18 (35.3%)	1 (33.3%	19 (34.5%)	–	–
Never	142 (62.8%)	71 (60.7%)	26 (59.1%)	1 (50.0%)	44 (62.0%)	–	71 (65.1%)	33 (64.7%)	2 (66.7%)	36 (65.5%)	–	–
ECOG performance status—no. patients (%)	–	–	–	–	–	0.69	–	–	–	–	0.87	0.14
0	32 (14.2%)	17 (14.5%)	5 (11.4%)	0	12 (16.9%)	–	15 (13.8%)	7 (13.7%)	1 (33.3%)	7 (12.7%)	–	–
1	190 (84.1%)	96 (82.1%)	38 (86.3%)	1 (50.0%)	57 (80.3%)	–	94 (86.2%)	44 (86.3%)	2 (66.7%)	48 (87.3%)	–	–
2	4 (1.7%)	4 (3.4%)	1 (2.3%)	1 (50.0%)	2 (2.8%)	–	0	0	0	0	–	–
Tumor histology—no. patients (%)	–	–	–	–	–	0.24	–	–	–	–	0.11	0.44
Adenocarcinoma	214 (94.7%)	109 (93.2%)	39 (88.6%)	2 (100%)	68 (95.8%)	–	105 (96.3%)	47 (91.2%)	3 (100%)	55 (100%)	–	–
Squamous cell carcinoma	11 (4.9%)	7 (6.0%)	4 (9.1%)	0	3 (4.2%)	–	4 (3.7%)	4 (7.8%)	0	0	–	–
Sarcoma	1 (0.4%)	1 (0.8%)	1 (2.3%)	0	0	–	0	0	0	0	–	–
Disease stage—no. patients (%)	–	–		–	–	0.57	–	–	–	–	0.34	0.22
IIIB/IIIC	10 (4.4%)	4 (3.4%)	1 (2.3%)	0	3 (4.2%)	–	6 (5.5%)	4 (7.8%)	0	2 (3.6%)	–	–
IV	216 (95.6%)	113 (96.6%)	43 (97.7%)	2 (100%)	68 (95.8%))	–	103 (94.5%)	47 (91.2%)	3 (100%)	53 (96.4%)	–	–
Liver metastasis at baseline—no. patients (%)	–	–	–	–	–	1	–	–	–	–	0.47	0.96
Yes	38 (16.8%)	21 (17.9%)	8 (18.2%)	0	13 (18.3%)	–	17 (15.6%)	9 (17.6%)	1 (33.3%)	7 (12.7%)	–	–
No	188 (83.2%)	96 (82.1%)	36 (81.8%)	0	58 (81.7%)	–	92 (84.4%)	42 (82.4%)	2 (66.7%)	48 (87.3%)	–	–
Brain metastasis at baseline—no. patients (%)	–	–	–	–	–	0.13	–	–	–	–	0.21	0.66
Yes	43 (19.0%)	21 (17.9%)	5 (11.4%)	0	16 (22.5%)	–	22 (20.2%)	8 (15.7%)	0	14 (25.5%)	–	–
No	183 (81.0%)	96 (82.1%)	39 (88.6%)	0	55 (77.5%)	–	87 (79.8%)	43 (84.3%)	0	41 (74.5%)	–	–
Methods used for evaluating brain metastasis—no. patients (%)	–	–	–	–	–	0.62	–	–	–	–	0.53	0.84
CT	10 (23.3%)	6 (28.6%)	1 (20.0%)	0	5 (31.3%)		4 (18.2%)	2 (25.0%)	0	2 (14.3%)	–	–
MRI	33 (76.7%)	15 (71.4%)	4 (80.0%)	0	11 (68.7%)		18 (81.8%)	6 (75.0%)	0	12 (85.7%)	–	–
*EGFR* mutation detection method—no. patients (%)	–	–	–	–	–	0.26	–	–	–	–	0.92	0.12
NGS	218 (96.5%)	115 (98.3%)	44 (100%)	2 (100%)	69 (97.2%)	–	103 (94.5%)	48 (94.1%)	3 (100%)	52 (94.5%)	–	–
ARMS-PCR	8 (3.5%)	2 (1.7%)	0	0	2 (2.8%)	–	6 (5.5%)	3 (5.9%)	0	3 (5.5%)	–	–
*EGFR* mutation at baseline form NGS (*n* = 218)—no. patients (%)	–	–	–	–	–	0.75	–	–	–	–	0.67	0.33
Exon 19 Del	124 (56.9%)	64 (55.6%)	23 (52.3%)	0	41 (57.7%)	–	60 (58.2%)	27 (52.9%)	0	33 (60.0%)	–	–
Exon 21 L858R	68 (31.2%)	40 (34.7%)	17 (38.6%)	0	23 (32.4%)	–	28 (27.1%)	14 (27.5%)	0	14 (25.5%)	–	–
Uncommon *EGFR* mutations	26 (11.9%)	11 (9.6%)	4 (9.1%)	2 (100%)	5 (7.1%)	–	15 (14.6%)	7 (13.7%)	3 (100%)	5 (9.1%)	–	–

ARMS-PCR, amplification-refractory mutation system-polymerase chain reaction; Bev, bevacizumab; CT, computed tomography; ECOG, Eastern Cooperative Oncology Group; ICI, immune checkpoint inhibitor; MRI, magnetic resonance imaging; NGS, next-generation sequencing.

a Cohort A vs. Cohort C.

b Cohort D vs. Cohort F.

### Treatment efficacy and progression-free survival

At data cutoff date (December 12, 2022), 100% (109/109) of patients who received Chemo/Chemo+Bev had events (disease progression or death) whereas only 95.7% (112/117) of patients who received Chemo+ICIs had events. Table 2 shows the distribution of the best overall response for patients in the Chemo/Chemo+Bev and Chemo+ICIs groups.

**Table 2:** Clinical outcomes in both cohorts

Objective response	Chemo+ICI (*n*, %)					Chemo/Chemo+Bev (*n*, %)					*p*^c^
	Total (*n* = 117)	Cohort A (*n* = 44)	Cohort B (*n* = 2)	Cohort C (*n* = 71)	*p*^a^	Total (*n* = 109)	Cohort D (*n* = 51)	Cohort E (*n* = 3)	Cohort F (*n* = 55)	*p*^b^	
PR	40 (34.2%)	19 (43.2%)	1 (50.0%)	20 (28.2%)	0.09	24 (22.0%)	7 (13.7%)	0	17 (30.9%)	0.03^d^	–
SD	54 (46.1%)	18 (40.9%)	1 (50.0%)	35 (49.3%)	0.38	53 (48.6%)	29 (56.9%)	3 (100%)	21 (38.2%)	0.05	–
PD	23 (19.7%)	7 (15.9%)	0	16 (22.5%)	0.39	32 (29.4%)	15 (29.4%)	0	17 (30.9%)	0.86	–
ORR	34.2%	43.2%	50.0%	28.2%	–	22.0%	13.7%	0	30.9%	–	0.04^d^
DCR	80.3%	84.1%	1 (100%)	77.5%	–	70.6%	70.6%	1 (100%)	69.1%	–	0.09

Bev, bevacizumab; DCR, disease control rate; ICI, immune checkpoint inhibitor; ORR, objective response rate, PD, progressive disease, PR, partial response, SD, stable disease.

a Cohort A vs. cohort C.

b Cohort D vs. cohort F.

c Chemo+ICI vs. Chemo/Chemo+Bev.

d Statistically significant *p* < 0.05. *p* values were computed by comparing the two groups using chi-square test.

**Figure 3:**
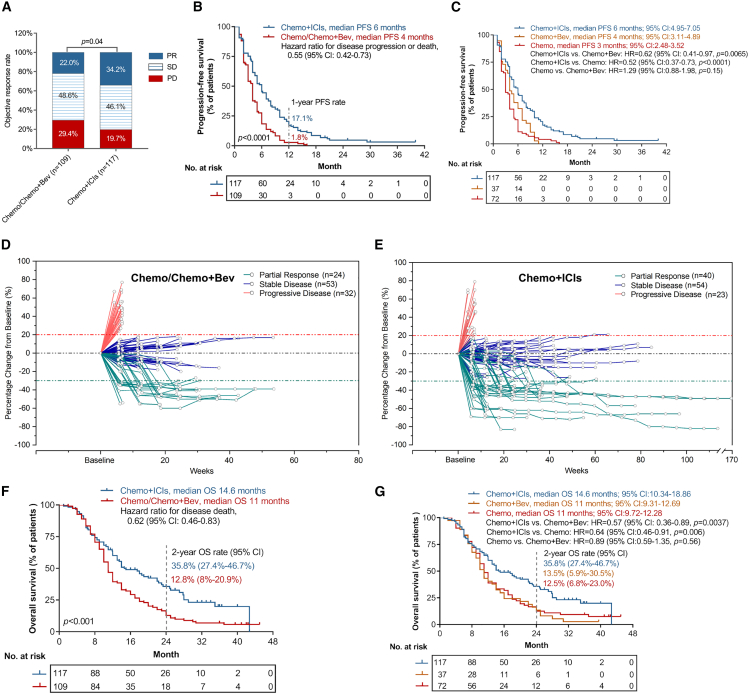
Treatment outcomes (A) Comparison of best objective response between patients who received chemotherapy with/without bevacizumab (Chemo/Chemo+Bev; *n* = 109) and chemotherapy plus immune checkpoint inhibitors (ICI) (Chemo+ICI; *n* = 117). PR, partial response; SD, stable disease; PD, progressive disease. (B and C) Kaplan-Meier survival analyses comparing the progression-free survival (PFS) of patients treated with either Chemo/Chemo+Bev or Chemo+ICI (B) or patients treated with either Chemo+ICI, Chemo+Bev, or Chemo (C). (D and E) Spider plots indicating the change in tumor size (relative to baseline) for each patient at specified time-points during treatment of Chemo/Chemo+Bev (D) or Chemo+ICI (E). (F and G) Kaplan-Meier survival analyses of overall survival (OS) for patients shown in (B and C) CI, confidence intervals; HR, hazard ratio.

According to the Response Evaluation Criteria in Solid Tumors (RECIST) criteria, the best overall response among 109 patients in the Chemo/Chemo+Bev group were as follows: 24 patients (22.0%) achieved partial response (PR), 53 patients (48.6%) had stable disease (SD), and 32 patients (29.4%) had progressive disease (PD). Contrastingly, the best overall response among 117 patients in the Chemo+ICIs group were as follows: 40 patients (34.2%) achieved PR, 54 patients (46.2%) had SD, and 23 patients (19.6%) had PD. The Chemo+ICI group had a significantly higher objective response rate (ORR) than the Chemo/Chemo+Bev group (34.2% vs. 22.0%; *p* = 0.04; Table 2; Figure 3A). Moreover, PFS was also significantly longer in the Chemo+ICIs group as compared with the Chemo/Chemo+Bev group (6.0 months vs. 4.0 months; *p* < 0.0001; Figure 3B). Further analysis showed that the difference in PFS still remained significant for Chemo+ICIs group when compared with the Chemo only group (without Bev) (6.0 months vs. 3.0 months; *p* < 0.0001; hazard ratio [HR]: 0.52 [95% confidence interval (CI): 0.37–0.73]) and Chemo+Bev group (6.0 months vs. 4.0 months; *p* = 0.007; HR: 0.62 [95% CI: 0.41–0.97]; Figure 3C).

The 1-year PFS probability was 17.1% for the Chemo+ICIs group and 1.8% for the Chemo/Chemo+Bev group. Additionally, the Chemo+ICIs group had a more remarkable extent of tumor shrinkage as compared with the Chemo/Chemo+Bev group (Figures 3D and 3E).

### Subgroup, univariate, and multivariate analyses for PFS

In the subgroup analysis, favorable PFS with Chemo+ICIs was seen across various baseline clinical, pathological, or molecular features demonstrated compared with Chemo/Chemo+Bev (Figure S1). Among patients who received Chemo+ICIs, no baseline clinicopathological features were associated with favorable PFS, including PD-L1 expression level, TMB, or the specific ICI that was administered (Figure S2). Univariate and multivariate analyses with the Cox proportional hazards regression model both indicated that receiving Chemo+ICIs after disease progression with EGFR-TKI was an independent predictor of better PFS as compared with receiving Chemo (without Bev) (univariate, *p* < 0.001; multivariate, *p* < 0.001) (Figure S3). Favorable PFS was observed for patients whose disease progressed with first-line, first-generation EGFR-TKI and subsequently received Chemo+ICIs as the second-line treatment (cohort D vs. cohort A; 6.0 months vs. 4.0 months; HR: 0.48 [95% CI: 0.31–0.75]; *p* < 0.001) (Figures S4 and S5A) or patients who progressed on the second-line, third-generation EGFR-TKI and subsequently received Chemo+ICIs as the third or subsequent-line therapy (cohort F vs. cohort C; 5.0 months vs. 3.6 months; HR: 0.54 [95% CI: 0.37–0.80]; *p* < 0.001) (Figures S4 and S5B).

### Overall survival

At the data cut-off date, patients who received Chemo+ICI had a median OS of 14.6 months, which was significantly longer as compared to patients who received Chemo/Chemo+Bev, who had a median OS of 11.0 months (Figure 3F). Moreover, the OS rate at two years was also significantly longer in the Chemo+ICIs group as compared to the Chemo/Chemo+Bev group (35.8% [95% CI: 27.4%–46.7%] vs. 12.8% [95% CI: 8.0%–20.9%]; *p* < 0.001; Figure 3F).

When OS were further analyzed based on treatment received, OS remained significantly longer in the Chemo+ICIs group compared with either the Chemo+Bev subgroup (14.6 months vs. 11.0 months; *p* = 0.004; HR: 0.57 [95% CI: 0.36–0.89]) or the Chemo subgroup (14.6 months vs. 11.0 months; *p* = 0.006; HR: 0.64 [95% CI: 0.46–0.91]; Figure 3G). Two-year OS rates were also longer for patients in the Chemo+ICIs group as compared to Chemo+Bev subgroup (35.8% [95% CI: 27.4%–46.7%] vs. 13.5% [95% CI: 5.9%–30.5%]) or the Chemo subgroup (35.8% [95% CI: 27.4%–46.7%] vs. 12.5% [95% CI: 6.8%–23.0%]; Figure 3G); however, the OS difference in Chemo+Bev vs. Chemo subgroups was not statistically significant (Figure 3G).

Consistently, the subgroup analysis demonstrated that Chemo+ICIs administered as second-line therapy was associated with a more favorable OS outcomes regardless of sex, smoking history, presence or absence of liver metastasis, and other variables analyzed (Figure S6). Univariate and multivariate analyses also demonstrated that the selection of treatment regimens had a significant impact on the OS, with patients treated with Chemo+ICIs exhibiting a better OS (univariate, *p* = 0.006; multivariate, *p* = 0.003; Figure S7).

The OS was also compared between different cohorts based on the generation of EGFR-TKIs administered previously (Figure S8). Among the patients who received first- or second-generation EGFR-TKI as first-line treatment, those who received Chemo+ICIs as the second-line treatment had a significantly longer OS than those who received Chemo/Chemo+Bev (cohort D vs. cohort A; 24.8 months vs. 13.0 months, HR: 0.45 [95% CI: 0.29–0.72]; *p* < 0.001; Figure S9A). Moreover, among patients who received third-generation EGFR-TKI as second- or later-line treatment, those who received Chemo+ICIs as the third-line or subsequent-line treatment tended to have a longer OS in comparison to those who received Chemo/Chemo+Bev, though not statistically different (cohort F vs. cohort C; 13.5 months vs. 10.0 months, *p* = 0.07; Figure S9B). Subgroup analyses also indicated that Chemo+ICI was associated with better survival outcomes as shown by the lower hazard ratio for both PFS and OS regardless of the EGFR-TKI history (Figures S4 and S8).

### Patient characteristics for constructing and validating the predictive model

In order to select the patients who can benefit from Chemo+ICI, we explored the utility of a non-invasive method as a potential predictor of treatment response. Baseline characteristics of the training and validation cohorts are listed in Table S1. Patients included in the training cohort were younger with a higher proportion of females and patients diagnosed with adenocarcinoma, which were consistent with classic clinical features of patients with *EGFR*-mutant NSCLC. Analysis of all lymphocyte subset profiles of the cohort revealed an abundance of T cells, followed by natural killer (NK) cells, and B cells. The clinical information and data on lymphocyte subsets from pre-treatment blood samples of every patient for each cohort are detailed in Data S1 (training cohort), Data S2 (validation cohort 1), and Data S3 (validation cohort 2).

### Constructing the LSM using the training cohort

**Figure 4:**
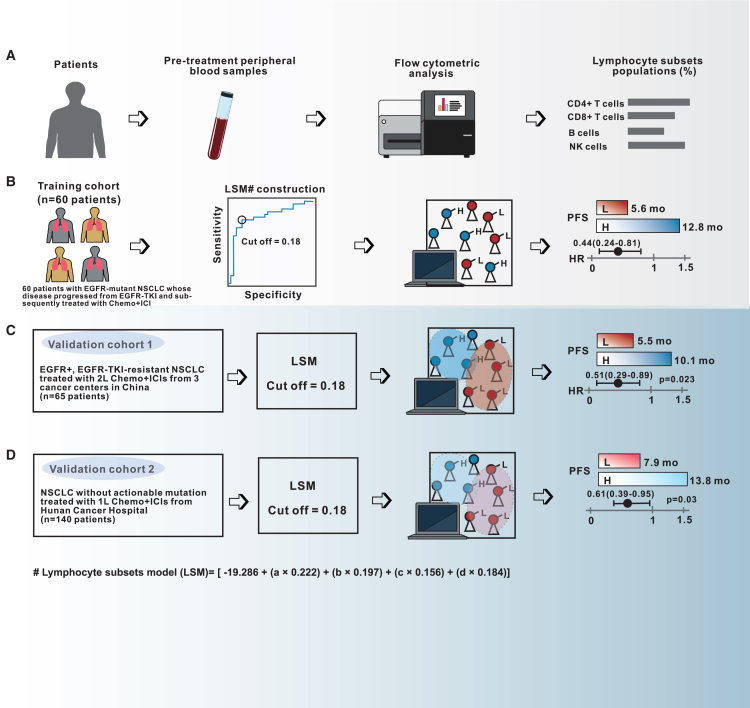
Schematic overview of the analytical and experimental design for the LSM Lymphocyte subsets model (LSM) is a potential non-invasive strategy in predicting treatment response and progression-free survival outcome with chemotherapy plus immune checkpoint inhibitors (Chemo+ICIs) in patients with advanced NSCLC. (A) Flow chart illustrating the study design. Pre-treatment peripheral blood samples are collected from the patients and are subjected to flow cytometric analysis to obtain the lymphocyte subsets data. (B) Training cohort refers to the 60 patients with *EGFR*-mutant NSCLC who received Chemo+ICI as subsequent-line treatment after EGFR-TKI progression. Data from the 60 patients’ baseline lymphocyte subset was used to construct the predictive model and determine the optimal cut-off for stratifying the patients based on LSM score as high and low. Treatment response and progression-free survival (PFS) outcome were compared between patients with LSM high and LSM low. LSM was validated using two independent cohorts as described in (C and D). (C) Validation cohort 1 refers to the independent cohort of 65 patients with *EGFR*-mutant NSCLC who received Chemo+ICI as second-line treatment after disease progression with EGFR-TKI from three cancer centers in China. (D) Validation cohort 2 refers to the independent cohort of 140 patients with NSCLC without actionable mutation who received Chemo+ICIs as first-line treatment from Hunan Cancer Hospital in China.

**Figure 5:**
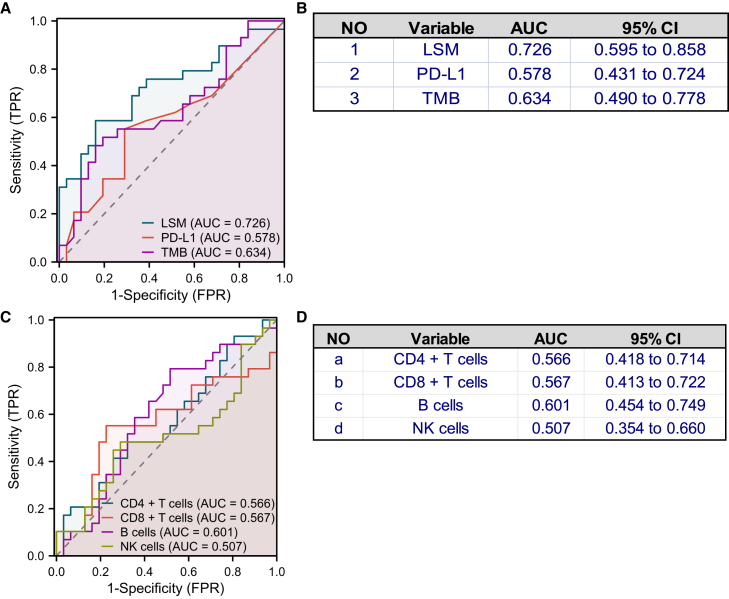
Predictive performance of the lymphocyte subset model (A) Receiver operating characteristic (ROC) curve illustrating the area under the curve (AUC) for comparing the performance in the binary classification of the training cohort using the lymphocyte subset model (LSM) model 1, PD-L1 expression and tumor mutation burden (TMB). The diagonal gray dashed line shows the line of best fit. FPR, false positive rate; TPR, true positive rate. (B) Table summarizing the AUC and corresponding 95% confidence intervals for the three biomarkers. (C) ROC curve illustrating the AUC for comparing the individual performance of each of the lymphocyte subsets (CD4^+^ T cells, A; CD8^+^ T cells, B; B cells, C; and NK cells, D) in predicting treatment outcomes with Chemo+ICI. (D) Table listing the AUC and corresponding 95% CI for each variable.

We sought to develop and validate the performance of a model based on lymphocyte subpopulation of CD4^+^ T cells, CD8^+^ T cells, B cells, and NK cells from flow cytometric analysis of pre-treatment blood samples of patients with EGFR-TKI-resistant, *EGFR*-mutant NSCLC for predicting the PFS outcomes with Chemo+ICI in both training and independent validation cohorts (Figure S10). Blood samples were collected at a median of 7 days (range: 2–15 days) after confirmation of disease progression and 12 days (range: 7–18 days) before treatment was administered. The schematic overview of the analytical workflow is presented in Figure 4. First, we utilized the logistic regression method to construct the lymphocyte subsets population model (LSM) by using *a*, *b*, *c*, and *d* values derived from the training cohort (*n* = 60), wherein *a* represents the CD4^+^ T cells (%), *b* represents the CD8^+^ T cells (%), *c* represents the B cells (%), and *d* represents the NK cells (%) (Figure 4A). Three LSM models were generated based on the probability of attaining a certain predefined clinical outcome (Figure S11). Given that 6.9 months was the reported median PFS for the combination therapy of Chemo+ICI plus IBI305 among patients with *EGFR*-mutated NSCLC who progressed on EGFR-TKIs in the ORIENT-31 study,^19^ we developed model 1 to predict the likelihood of achieving a PFS of ≥9.0 months (using the median PFS as the positive predictive value) with Chemo+ICIs. The 60 patients in the training cohort were stratified into two subgroups based on PFS ≥9.0 months and <9.0 months to construct the model and to evaluate its performance in classifying patients with PFS ≥9.0 months from patients with PFS <9.0 months. This model had an area under the curve (AUC) of 0.726. Similarly, we built model 2 to predict the likelihood of achieving a PFS of partial response, which yielded an AUC of 0.606. Model 3 was built to predict the likelihood of disease progression, which had an AUC of 0.610. Based on AUC values, we then selected model 1 as the final LSM for subsequent analyses, which had a sensitivity of 58.6% and a specificity of 83.9% (Figure 5A). The formula of this model is as follows: LSM = [−19.286 + (*a* × 0.222) + (*b ×* 0.197) + (*c ×* 0.156) + (*d ×* 0.184)]. The receiver operator characteristic (ROC) yielded a Youden index of 0.42, wherein an optimal cut-off of 0.18 was selected.

We also compared the predictive performance of PD-L1 expression, TMB, and the four lymphocyte subtypes when used individually to predict PFS outcomes. These biomarkers showed a poorer predictive ability when used individually, with the AUC ranging between 0.507 and 0.634 (Figures 5A–5D). Thus, our findings suggested that combining various biomarkers such as values for *a*, *b*, *c*, and *d* in the LSM had a synergistic effect, which generally improved its predictive ability. Combined analysis of two or more biomarkers, including PD-L1 plus LSM and PD-L1 (AUC = 0.749), PD-L1 plus LSM (AUC = 0.735), TMB plus LSM (AUC = 0.735), demonstrated higher predictive performance than LSM alone (AUC = 0.726) (Figure S12).

### Comparison of PFS between the LSM-high and LSM-low subgroups for training and two independent validation cohorts

The median PFS was 9.0 months (95% CI: 6.6–11.4) for the training cohort, 6.6 months (95% CI: 5.2–8.1) for the validation cohort 1, and 8.5 months (95% CI: 6.0–11.0) for the validation cohort 2 (Figure S13).

**Figure 6:**
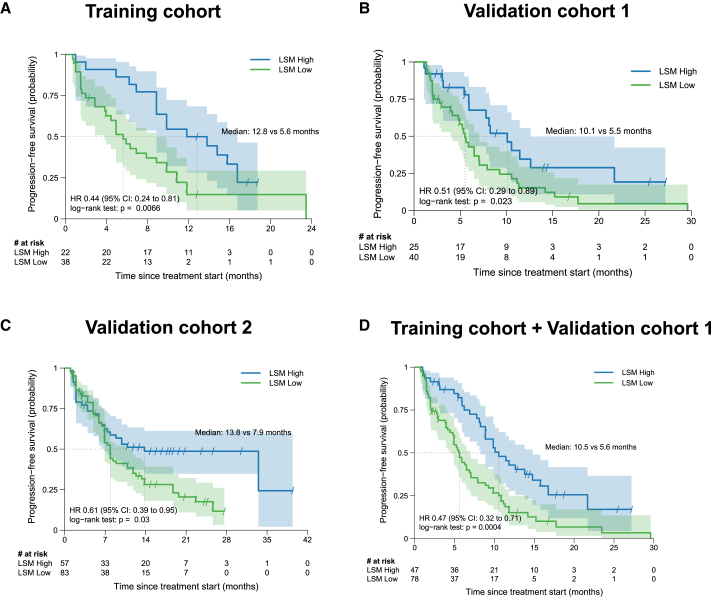
Lymphocyte subset model validated in two independent NSCLC cohorts (A–C) Kaplan-Meier survival curves comparing the progression-free survival (PFS) between lymphocyte subset model (LSM)-high and LSM-low subgroups for the training cohort (A), validation cohort 1 (B) and validation cohort 2 (C). CI, confidence intervals; HR, hazard ratio.

The training cohort was stratified into two subgroups as LSM-high (*n* = 38) and LSM-low (*n* = 22) using the cut-off of 0.18. As expected, compared with the LSM-low subgroup, patients in the LSM-high subgroup had a significantly longer PFS (median, 12.8 months vs. 5.6 months; *p* = 0.007; HR = 0.44 [95% CI: 0.24–0.81]; Figure 6A). We found no statistical difference in PFS between subgroups with different PD-L1 expression levels (<1% vs. ≥ 1% and <50% vs. ≥ 50%) and TMB (≤4.8 vs. > 4.8 mutations/Mb) (Figure S14).

To further validate the generalizability of the LSM constructed based on the training cohort, we retrieved data from two independent cohorts. As described in the STAR Methods section, the validation cohort 1 composed of 65 patients with EGFR-TKI-resistant, *EGFR*-mutated NSCLC from three different cancer centers and the validation cohort 2 composed of 140 patients pooled together from Hunan Cancer Hospital who had NSCLCs that lacked actionable genetic mutations and received ICI-based regimens as first-line therapy in the real-world setting. Using the same cutoff value of 0.18, we stratified these patients into the LSM-high and LSM-low subgroups. Consistently, the analyses also revealed that patients in the LSM-high subgroup had significantly longer PFS outcomes compared to those in the LSM-low subgroup in both validation cohorts 1 and 2 (Figures 6B and 6C). Univariate and multivariate analyses with the Cox proportional hazards regression model in the training cohort and validation cohorts 1 and 2 indicated that having LSM-high, but not PD-L1-high and TMB-high levels, was an independent predictor of a better PFS with Chemo+ICI therapy (Figures S15–S17).

Using the training dataset, each of the four lymphocyte subsets showed minimal collinearity (Table S2). Consistently, univariate analysis showed that each of the four lymphocyte subsets had no impact on PFS (Figure S18). LSM-high subgroup had favorable outcomes regardless of PD-L1 expression and TMB status (Figure S19).

**Figure 7:**
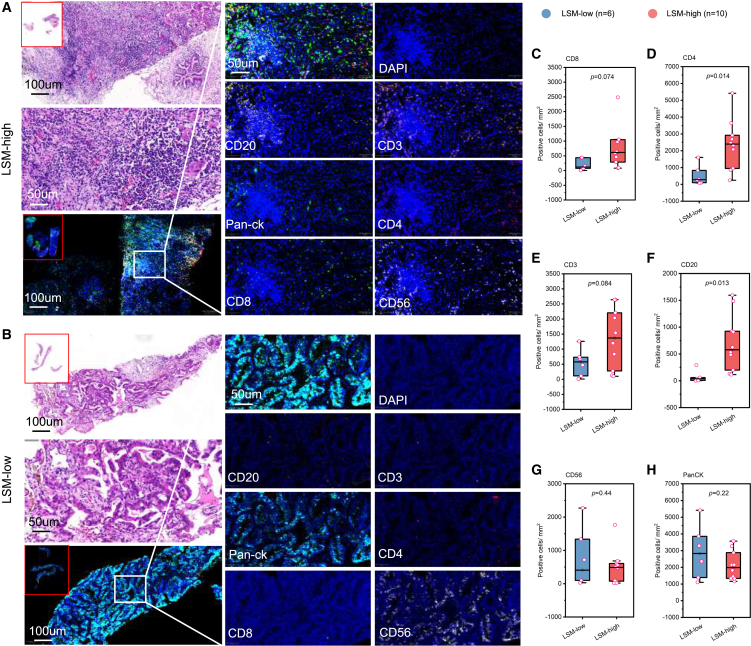
Distinct tumor immune microenvironment of patients with EGFR-TKI-resistant NSCLC with LSM-high (A and B) Representative micrographs for hematoxylin-eosin and fluorescence immunohistochemistry assay for CD20, CD3, pan-cytokeratin (CK), CD4, CD8, and CD56 for a patient with LSM-low (A) and LSM-high (B). All microscopy images include scale bars of 100 μm and 50 μm, as indicated. (C–H) Boxplots comparing the distribution of positive cell/mm^2^ between LSM-low (*n* = 6) and LSM-high (*n* = 10) subgroups for CD8 (C), CD4 (D), CD3 (E), CD20 (F), CD56 (G), and pan-CK (H) in patients with EGFR-TKI-resistant NSCLC. Statistical comparisons were performed using t test. The box represents the interquartile range, with the line inside indicating the median value. Each dot marks the individual data point, and dots located outside the whiskers represent outliers. The top and bottom edges of the box correspond to the lower and upper quartiles, while the whiskers extending from the box show the minimum and maximum values in the dataset.

Lastly, we performed multiplex immunofluorescence assay to quantify the protein expression of these lymphocyte subsets in the LSM-low and LSM-high subgroups in patients with EGFR-TKI-resistant NSCLC (Figures 7A–7F). Compared with the LSM-low subgroup, the LSM-high subgroup had a significantly higher expression of CD4^+^ T cells (*p* = 0.014; Figure 7D) and CD20^+^ B cells (*p* = 0.013; Figure 7F).

Analysis of molecular resistance mechanisms of pre-treatment samples shows no difference between patients in the training cohort and validation cohort (Figure S20) as well as between LSM-high and LSM-low subgroups from the training and validation cohorts (Figure S21).

## Discussion

In this retrospective study, we first evaluated the effectiveness of Chemo+ICI in comparison with platinum-based chemotherapy (with or without Bev) for patients with *EGFR*-mutated, EGFR-TKI-resistant NSCLC in the real-world clinical setting. Subsequently, we sought to identify a non-invasive, blood-based predictive biomarker that enables the selection of the subset of patients who will more likely benefit from ICI-containing regimen. Our study provides meaningful clinical evidence demonstrating the superiority of the combination therapy of Chemo+ICI in terms of significantly prolonging PFS and OS as compared to chemotherapy (with or without Bev) based on a large patient cohort from a real-world setting.

*EGFR*-mutant NSCLCs account for half of the patients with NSCLCs diagnosed in East Asia. Despite the superior outcomes with EGFR-TKIs, disease progression inevitably develops, with 20%–58% of patients whose tumors lack actionable mutations and receiving chemotherapy as subsequent-line therapy.^7^ Preclinical studies have shown that EGFR signaling exerts an immunomodulatory role by regulating MHC I/II and PD-L1 expression on tumor cells and lymphocyte activity.^28^ The microenvironment for *EGFR*-mutated tumors is immunosuppressive, and EGFR-TKIs can regulate tumor immune microenvironment by affecting the expression of tumor cell antigens and infiltration of immune cells.^29^ It has been shown that patients with *EGFR*-mutated tumors having a higher PD-L1 expression levels were less likely to benefit from EGFR-TKI treatment.^30^ Therefore, it seems feasible to include PD-1/PD-L1 inhibitors in the later-line treatment of patients with *EGFR*-mutated NSCLC. Despite prior evidence showing that *EGFR*-mutated NSCLCs were less responsive to PD-L1/PD-1 inhibitor monotherapy, which was similarly shown by the final results from the CheckMate 722 and KEYNOTE-789,^16^^,^^17^^,^^31^^,^^32^^,^^33^^,^^34^ recent clinical data are now suggesting a potential role of ICI-based combination therapies in the subsequent-line treatment of this patient subset.^18^^,^^19^ Subgroup analysis and preliminary findings from phase 3 randomized controlled trials, including IMpower150, ORIENT-31, and ATTLAS, suggested that a four-drug regimen combining PD-L1/PD-1 monoclonal antibody plus Bev plus chemotherapy might represent a potential therapeutic option for patients with *EGFR*-mutated, EGFR-TKI-resistant NSCLCs.^18^^,^^35^^,^^36^ The IMpower150 trial reported improved OS for patients treated with Bev/carboplatin/paclitaxel plus atezolizumab (ABCP) versus Bev/carboplatin/paclitaxel (BCP), and revealed comparable survival outcomes for patients treated with atezolizumab/carboplatin/paclitaxel or BCP; however, that analysis was simply exploratory and inadequately powered to draw definitive conclusions.^18^ In the IMpower151 study, a comparable investigator-assessed median PFS was seen between Chinese patients with *EGFR*/*ALK*-mutated NSCLC who received ABCP and those who received BCP (8.5 months vs. 8.3 months; HR = 0.86 [95% CI: 0.61–1.21]).^37^ Contrastingly, the ATTLAS study demonstrated significantly longer PFS in patients with *EGFR*/*ALK*-mutated NSCLC who received ABCP than BCP (8.5 months vs. 5.6 months; HR = 0.62 [95% CI: 0.45–0.86]; *p* = 0.004).^36^ Moreover, the ORIENT-31 trial demonstrated the superiority of the four-drug strategy with sintilimab plus Bev biosimilar IBI305 and platinum-based chemotherapy over chemotherapy alone, but it did not include any comparison of treatment outcomes with control groups treated with three-drug regimens, such as Chemo+ICI or Chemo+Bev.^35^ The ORIENT-31 study have shown a significantly longer PFS in patients who received sintilimab plus chemotherapy vs. chemotherapy only (arm B vs. arm C; 5.5 months [95% CI: 4.5–6.1]vs. 4.3 months [95% CI: 4.1–5.3]; HR = 0.72 [95% CI: 0.55–0.94], *p* = 0.016).^35^ Although the findings from these prospective randomized controlled studies were encouraging and have partly addressed some questions with respect to the efficacy of ICI-containing regimen as subsequent-line treatment of *EGFR*-mutated EGFR-TKI-resistant NSCLCs, more clinical data are required to yield more definitive insights on the specific combination regimen that could afford optimal clinical outcome, given the lack of data on head-to-head comparisons between these regimens (i.e., ICIs plus Bev and chemotherapy, ICIs plus chemotherapy, or Bev plus chemotherapy).

Instead of a four-drug combination regimen (i.e., ICI + Bev + platinum-based chemotherapy), a three-drug combination regimen of Chemo+ICIs could serve as an alternative treatment option for patients with *EGFR*-mutated, EGFR-TKI-resistant NSCLC, particularly for those who were unable to tolerate Bev due to adverse events such as hypertension and bleeding risk. In our study, the ORR in the Chemo+ICIs group reached 34.2% and median OS reached 14.6 months. Except for the economic benefits and the advantage of reducing Bev-related adverse events, our study demonstrated that Chemo+ICIs regimen is more effective than Chemo+Bev, leading to a long-term survival benefit for patients with EGFR-TKI pretreated, *EGFR*-mutated NSCLC. Additionally, the two-year OS rate in the Chemo+ICIs group was significantly higher than the Chemo/Chemo+Bev group, and the 95% CIs were not overlapped, indicating the absolute survival predominance of patients who received Chemo+ICIs. Our findings corroborated the encouraging findings of the ORIENT-31, the IMpower150, and the ATTLAS study.^18^^,^^19^^,^^36^

Several studies evaluated the efficacy of Chemo+ICIs. For instance, a phase 2 study suggested that pembrolizumab combined with chemotherapy demonstrated an ORR of 42%, a median PFS of 8.3 months, and a median OS of 22.2 months among patients with recurrent *EGFR*-mutated NSCLC (*n* = 26).^38^ Another retrospective study with a smaller cohort of 144 patients reported a comparable PFS but higher ORR for ICI-containing combination regimens as subsequent-line therapy in pretreated, *EGFR*-mutant NSCLC.^39^ Our study provided additional real-world insights into improvements in both PFS and OS for patients treated with Chemo+ICI compared with those treated with Chemo/Chemo+Bev. Taken together, we propose Chemo+ICIs as an alternative treatment strategy for patients with *EGFR*-mutated EGFR-TKI-resistant NSCLCs, highlighting its effectiveness, safety, and pharmaco-economic concerns.

Besides patients’ archived tumor tissue samples, blood samples are biological specimens that are readily accessible in a clinical setting for various clinical and exploratory assays. Lymphocyte infiltration is associated with ICI efficacy.^40^ Previous studies reported that T cells, B cells, and NK cells are three main types of immune cells involved in controlling anti-tumor immunity.^41^^,^^42^ A previous study indicated that T lymphocyte subsets could be a potential indicator of immune function in patients with lung cancer.^43^ Another study reported that changes in lymphocyte subsets before and after radiotherapy, especially blood lymphocyte count and CD4^+^ T cells, were associated with survival outcomes of patients with NSCLC.^44^ These studies suggested that blood lymphocytes might be a promising biomarker of ICI efficacy. In our present study, we explored the utility of combining the data from proportions of CD4^+^ T cells, CD8^+^ T cells, B cells, and NK cells. These biomarkers showed poor predictive ability when used individually; however, their combination synergistically improved the predictive performance of the LSM in terms of distinguishing the subgroup of patients who would have favorable treatment outcomes with Chemo+ICIs. Despite the higher AUC for combined analysis of LSM, PD-L1 expression and TMB, the LSM-high subgroup had favorable PFS outcome regardless of PD-L1 expression and TMB status. This finding raises the potential utility of LSM as an effective predictive biomarker. Moreover, LSM was consistently effective in identifying the subset of patients with advanced NSCLC who could benefit from Chemo+ICIs, not only as subsequent-line therapy of *EGFR*-mutant NSCLCs after developing disease progression with EGFR-TKI, but also as first-line treatment of NSCLCs without actionable mutations. Flow cytometric analysis of lymphocyte subsets based on patients’ whole blood samples is clinically practical, easily accessible, time and cost-effective (less than US$100 per assay). Our findings demonstrated that LSM could serve as a novel, routine, cost-effective, and non-invasive approach for identifying the subset of patients who are more likely to benefit from Chemo+ICIs, regardless of the NSCLC subtype. Our findings are hypothesis-generating and highlight the need for further clinical studies to validate the effectiveness of this biomarker combination. Additionally, deeper investigation into the biological mechanisms underlying LSM is warranted, including single-cell transcriptomic profiling, functional assays, and advanced immune phenotyping to delineate the roles of individual lymphocyte subsets and their interactions and synergy.

Our study provides real-world evidence demonstrating that Chemo+ICI as subsequent-line therapy can significantly prolong PFS and OS of *EGFR*-mutant EGFR-TKI resistant NSCLCs compared to standard chemotherapy with or without Bev. Furthermore, our study explores the potential of a flow cytometry-based analysis of pre-treatment whole blood lymphocyte subsets in predicting treatment outcomes with Chemo+ICIs for patients with advanced NSCLC, irrespective of treatment history and *EGFR* mutation status. While these findings suggest LSM may serve as a promising tool for patient selection, its predictive value requires further validation in prospective studies. Additional research is necessary to refine and validate this approach before considering its clinical applicability.

### Limitations of the study

Our study has several limitations associated with retrospective studies. First, this study’s retrospective nature introduces potential selection bias, where patients included in this study were previously treated with varied treatment histories rather than randomization. Variability in resistance mechanisms may influence treatment outcomes. Nevertheless, Chemo+ICIs was associated with improved survival outcomes, regardless of previous EGFR-TKIs exposure. To validate and extend the utility of the LSM across broader patient populations, a prospective study with a larger sample size is needed. Second, incomplete data on PD-L1 expression and TMB limited their use as benchmarks for LSM due to the lack of tissue samples. The lack of comprehensive PD-L1 information may have influenced treatment decisions, particularly for those receiving chemotherapy alone, and highlights the need for further investigations into how PD-L1 status may be integrated with LSM predictions. Despite these limitations, our findings provide preliminary evidence supporting LSM as a promising predictive biomarker for Chemo+ICI. As a blood-based biomarker, LSM offers practical advantages over tumor biopsy-dependent PD-L1 or TMB assessment, improving accessibility and cost-effectiveness. Third, the time gap between blood collection for LSM and treatment initiation may not be uniform across the study cohort. Given potential fluctuations in lymphocyte subsets over time, this delay may impact LSM’s predictive accuracy. Further research is needed to assess immune profile stability and determine optimal timing for biomarker evaluation. Fourth, while most tumor specimens underwent NGS analysis, inconsistencies in targeted panel selection and sequencing depth hindered precise TMB estimation. Fifth, the relatively small sample size in validation cohort 1 may also introduce bias, though consistent findings across training and validation cohorts (both 1 and 2) suggest LSM’s potential applicability for patient selection in NSCLC irrespective of treatment line. Its utility in other tumor types receiving ICIs as first- or second-line therapies warrants further exploration. Lastly, blood and tissue samples were not collected to characterize the tumor microenvironment of patients with actionable driver-negative NSCLC who received Chemo+ICI as first-line treatment (validation cohort 2). Investigating immune differences between *EGFR*-mutant and *EGFR* wild-type NSCLC remains an important avenue for future research.

## Resource availability

### Lead contact

Further information and requests for resources should be directed to and will be fulfilled by the lead contact, Yongchang Zhang (zhangyongchang@csu.edu.cn).

### Materials availability

This study did not generate new unique reagents.

### Data and code availability

• All somatic mutation status analyzed in this study was extracted from patients’ electronic medical records.
• No DNA sequencing analysis or original code development was performed as part of this work.
• The patient data supporting the findings of this study are provided in Data S1, S2, and S3.
• Any additional information required to reanalyze the data is available from the lead contact upon request.

## Acknowledgments

We thank Dr. Analyn Lizaso for her editing assistance. This work was financially supported by the National Natural Science Foundation of China (grant numbers: 82222048, 82003206, 82173338, 82102747, and 82160489), the Natural Science Foundation of Hunan Province (grant numbers: 2021RC4040, 2023JJ30371, and 2023JJ30368), and School-College Joint Foundation of Hunan University of Chinese Medicine (grant number: 2024XYLH269). The funding agencies had no role in the study design, data collection, analysis, interpretation, manuscript writing, and the decision to submit the article for publication.

## Author contributions

Y.Z. and N.Y., conceptualization, organization, data collection, auditing, supervision, project management, funding acquisition, and writing – review and editing; L. Zeng. and L.S., data curation, methodology, formal analysis, original draft preparation, and writing – review and editing; Z.H., F.T., Z.L., and Q.X., software operation, data validation, and writing – review and editing; L.S., H.Q., H.Y., X.Z., Z.H., J.Y., X.Y., Y.X., Z.W., L. Zhang., and L.D., formal analysis, visualization, and writing – review and editing; N.Y., W.G., S.L., and X.D., critical comments and suggestions, and writing – review and editing.

## Declaration of interests

The authors declare no competing interests.

## STAR★Methods

### Key resources table

**Table:** 

REAGENT or RESOURCE	SOURCE	IDENTIFIER
**Antibodies**		

anti-CD3-PerCP	BD Biosciences	Cat# 555329; RRID: AB_395736
anti-CD4-FITC	BD Biosciences	Cat# 555346; RRID: AB_395751
anti-CD8-PE	BD Biosciences	Cat# 560179; RRID: AB_1645481
anti-CD19-APC	BD Biosciences	Cat# 555415; RRID: AB_398597
anti-CD16-PE	BD Biosciences	Cat# 557758; RRID: AB_396864
anti-CD56-PE	BD Biosciences	Cat# 555513; RRID: AB_395903
PD-L1 22C3	Agilent PharmDx	SK006; RRID: AB_2889976
Human monoclonal DAPI	Abcam	Cat# ab104139; RRID: AB_2629482
Human monoclonal anti-CD3	Abcam	Cat# ab16669; RRID: AB_2884903
Human monoclonal anti-CD4	Abcam	Cat# ab133616; RRID: AB_2750883
Human monoclonal anti-CD20	Abcam	Cat# ab46895; RRID: AB_726233
Human monoclonal anti-CD56	Abcam	Cat# ab119587; RRID: AB_2935137
Human monoclonal anti-panCK	Abcam	Cat# ab7753; RRID: AB_306047

**Biological samples**		

Human archived tissue specimen	This study	N/A
Human blood samples	This study	N/A

**Critical** commercial **assays**		

168-gene panel	Burning Rock Biotech	
Amplification-refractory mutation system polymerase chain reaction	Amoy Diagnostics	

**Software and** algorithms		

Cell Quest Pro software	BD Biosciences	
R (version 3.3.3)	R Foundation for Statistical Computing	
R Studio (version 1.1.383)	R Foundation for Statistical Computing	

**Other**		

Pembrolizumab	Merck Sharp and Dohme	N/A
Sintilimab	Eli Lilly and Co/Innovent Biologics	N/A
Toripalimab	Coherus BioSciences	N/A
Tislelizumab	BeiGene	N/A
Atezolizumab	Roche	N/A
Pemetrexed	Eli Lilly/Qilu Pharmaceuticals	N/A
Paclitaxel	Qilu Pharmaceuticals	N/A
Carboplatin	Qilu Pharmaceuticals	N/A
Carboplatin	Qilu Pharmaceuticals	N/A
Bevacizumab	Roche	N/A

### Experimental model and study participant details

#### Study design and patients included in the study

We retrospectively analyzed clinical data from two groups of patients with advanced NSCLC whose tumors lacked actionable mutations. We screened 1,647 patients whose tumor samples were subject to molecular testing of *EGFR* mutations between January 1, 2018 and October 1, 2022. This patient cohort was from Hunan Cancer Hospital. 226 patients who received subsequent-line therapy as either ICI plus chemotherapy regimen (Chemo+ICI) or chemotherapy regimen with or without bevacizumab (Chemo/Chemo+Bev) after EGFR-TKI progression were included in this study. These patients were further stratified into two subgroups based on the treatment regimen they received upon disease progression with EGFR-TKI. Patients who received Chemo+ICIs were grouped together and patients who received Chemo/Chemo+Bev were grouped separately. For further subgroup analyses, these two groups were further stratified into Cohorts A-F according to the treatment-line (first- or second line) and generation of EGFR-TKIs (first-, second- or third-generation) from prior regimen. We compared the clinical effectiveness of ICI-based regimen and chemotherapy regimen for each subgroup. Participant information on sex, age, and ethnicity was self-reported and recorded in the electronic medical records. Information on gender was not collected. The approval for the study protocol was obtained from the Institutional Ethics Committee of Hunan Cancer Hospital. Written informed consent was waived due to the retrospective nature of this study.

#### Criteria of patient inclusion

Criteria of patient inclusion in our study were as follows: (1) histologically confirmed as advanced or metastatic NSCLC; (2) positive detection of sensitizing *EGFR* mutations either by amplification-refractory mutation system polymerase chain reaction (ARMS-PCR) or NGS using a 168-gene panel (Burning Rock Biotech, Guangzhou, China); (3) confirmed disease progression after receiving first-, second- or third-generation EGFR-TKI therapy; and (4) no actionable mutations after disease progression with EGFR-TKI. Tumor samples from patients who progressed on either first- or second-generation EGFR-TKI should be negative for *EGFR* T790M mutation; otherwise, they should be confirmed to have disease progression with third-generation EGFR-TKIs. Patients who received third-generation EGFR-TKI as first-line therapy were included regardless of their *EGFR* T790M mutation status.

### Method details

#### Efficacy and safety evaluation

ICIs administered included pembrolizumab and sintilimab (200 mg, intravenously every 21 days). Response was assessed according to the Response Evaluation Criteria in Solid Tumors (RECIST, version 1.1). Tumor assessment was performed at baseline prior to treatment (Day 0) and after every 2 cycles for 6–8 weeks until confirmation of disease progression. Treatment response was assessed using computed tomography (CT) or magnetic resonance imaging (MRI). The overall response rate (ORR) was defined as the proportion of patients achieving partial response (PR) or complete response (CR). PFS was calculated as the time interval from initiating treatment following EGFR-TKI progression until confirmation of tumor progression, death from any cause, or the last date of follow-up. Overall survival (OS) was defined as the time interval between initiating treatment following EGFR-TKI progression until death or last follow-up. Every systemic response evaluation and each radiological image was independently evaluated by the physician-in-charge and two radiologists, respectively.

#### Patients included and study design for constructing the LSM model

This study retrospectively analyzed data from a total of 270 patients with locally advanced/advanced NSCLCs who received Chemo+ICI treatment and had whole blood lymphocyte subsets data before receiving the regimen. The training cohort was comprised of 60 EGFR-TKI pre-treated patients with *EGFR*-mutant NSCLC who received Chemo+ICI regimen after disease progression with EGFR-TKI between January 1, 2018, and September 30, 2021 from Hunan Cancer Hospital. The inclusion criteria were as follows: (1) histologically confirmed advanced or metastatic NSCLC; (2) confirmed disease progression after receiving first-, second- or third-generation EGFR-TKI therapy; and (3) no actionable mutations after disease progression with EGFR-TKI. Patients who progressed from either first- or second-generation EGFR-TKI should be negative for *EGFR* T790M mutation; otherwise, they should be confirmed to have disease progression from third-generation EGFR-TKIs. Patients who received third-generation EGFR-TKI as first-line therapy were included regardless of their *EGFR* T790M mutation status. The validation cohort 1 comprised of 65 patients with EGFR-TKI-resistant, *EGFR*-mutant NSCLC who received Chemo+ICI after disease progression with first-line EGFR-TKI from three different cancer centers, including the Second Affiliated Hospital, Zhejiang University School of Medicine, Cancer Center, Union Hospital, Tongji Medical College, Huazhong University of Science and Technology, and Peking University Cancer Hospital. The validation cohort 2 included 140 treatment-naïve patients diagnosed with NSCLC without actionable mutation who received Chemo+ICI as first-line therapy in a real-world setting from Hunan Cancer Hospital in China (Figure S10).

#### Treatment regimen and evaluation of efficacy

Chemotherapy regimens were administered as platinum-based doublet regimens in combination with paclitaxel, nab-paclitaxel, docetaxel, or pemetrexed. For the training cohort, ICIs included the PD-1 inhibitor pembrolizumab, sintilimab, tislelizumab and toripalimab, and the PD-L1 inhibitor atezolizumab (200 mg, intravenously every 21 day). ICI regimens in validation cohort 1 was similar to the training cohort. In the validation cohort 2, ICIs were administered according to the selection of patients, which included sintilimab, pembrolizumab, and toripalimab. Treatment response was assessed by the physician-in-charge according to the RECIST version 1.1. Tumor assessment was performed at baseline before treatment at Day 0 and after treatment every 2 cycles for 6–8 weeks until disease progression was confirmed. Treatment response was assessed using CT or MRI. ORR was defined as the proportion of patients PR and CR. Disease control rate referred to the proportion of patients who achieved CR, PR and stable disease (SD) as the best objective response. PFS was calculated as the time interval from initiation of Chemo+ICI treatment until the confirmation of tumor progression, death from any cause, or the last date of follow-up. OS was defined as the time interval between initiating treatment until death or the last follow-up date. Every systemic response evaluation and each radiological image was independently evaluated by the physician-in-charge and two radiologists.

#### Flow cytometric analysis of lymphocyte subsets from pretreatment whole blood samples

To identify the pretreatment status of lymphocyte subsets, peripheral blood samples collected before administration of treatment regimens were subjected to flow cytometric analysis. The following anti-human monoclonal antibodies were used: anti-CD3-PerCP, anti-CD4-FITC, anti-CD8-PE, anti-CD19-APC, and anti-CD16^+^CD56-PE (BD Biosciences, Multitest IMK Kit, BD Biosciences, New Jersey, USA). 50 μL aliquot of blood samples collected in EDTA-containing tubes were incubated with the appropriate antibody cocktails for 30 min on ice, washed twice in phosphate-buffered saline, and fixed with 1% paraformaldehyde before flow cytometric analysis using a Fluorescence-activated cell sorter (FACS) Calibur cytometer with Cell Quest Pro software (BD Biosciences, New Jersey, USA). At least 10,000 cells were collected in the lymphocyte gate and subsequently analyzed. T lymphocytes were identified (CD3^+^), and further stratified into CD4^+^ or CD8^+^ subpopulations. Numbers and percentages of B lymphocytes (CD3^−^ CD19^+^) and natural killer (NK) cells (CD3^−^ CD56^+^ CD16^+^) were also determined.

#### LSM construction

The LSM was constructed based on the logistic regression model using the data of four lymphocyte subsets, wherein *a* represents the CD4^+^ T cells (%), *b* represents the CD8^+^ T cells (%), *c* represents the B cells (%), and *d* represents the NK cells (%). The LSM was constructed to predict the treatment outcome (i.e., PFS over 9.0 months, achieving PR as best response, or experiencing PD at cut-off date). The magnitude of the coefficients reflects the strength of the independent variable’s influence on the dependent variable. A larger coefficient indicates a greater impact of the independent variable on the dependent variable. We first recognize that the effect of the immune microenvironment on ICIs is highly complex. CD4^+^ T cells, CD8^+^ T cells, B cells, and NK cells all contribute to the effectiveness of immunotherapy. Different coefficient values represent the varying degrees of involvement of each cell type in the immune microenvironment. Among them, CD4^+^ T cells and CD8^+^ T cells play a central role in cellular immune regulation. The receiver operator characteristic (ROC) curve method was used to determine the predictive performance of the model. The Youden index was used to select the optimal cut-off value.

### Quantification and statistical analysis

#### Statistical analysis

Continuous variables were summarized as means and standard deviations or medians with range and compared using unpaired t-test or Wilcoxon signed-rank test. Categorical variables were summarized as frequencies with percentages and compared using Chi-square or Fisher’s exact test, as appropriate. Kaplan-Meier analysis was used to estimate the survival functions and log-rank test to determine the difference in survival outcomes between groups. The Cox proportional hazards model was used for multivariate survival analysis. Schoenfeld residuals were used to check the proportional hazards assumption. All tests were two-sided, and *p* value <0.05 was considered statistically significant. All statistical analyses were performed using R (version 3.3.3, the R Foundation for Statistical Computing, Vienna, Austria) and R Studio (version 1.1.383).

## Supplemental information

Document S1. Figures S1–S21 and Tables S1 and S2

Data S1. Clinical information and data on lymphocyte subsets from pre-treatment blood samples of the training cohort

Data S2. Clinical information and data on lymphocyte subsets from pre-treatment blood samples of the validation cohort 1

Data S3. Clinical information and data on lymphocyte subsets from pre-treatment blood samples of the validation cohort 2
