# Supplementary material for: Lymphocyte subset-based non-invasive biomarker predicts immunochemotherapy efficacy in EGFR-TKI-pretreated EGFR-mutated NSCLC
Source: iScience. 2025 Jul 25;28(9):113211. doi: 10.1016/j.isci.2025.113211 (PMC12358657; doi:10.1016/j.isci.2025.113211)
Supplement: Document S1. Figures S1–S21 and Tables S1 and S2 [file mmc1.pdf]

iScience, Volume *28*

Supplemental information

Lymphocyte subset-based non-invasive biomarker predicts immunochemotherapy efficacy in EGFR-TKI-pretreated EGFR-mutated NSCLC

Lianxi Song, Liang Zeng, Qinqin Xu, Yizhi Li, Wenhuan Guo, Shaoding Lin, Wenjuan Jiang, Zhan Wang, Li Deng, Zhe Huang, Haoyue Qin, Huan Yan, Xing Zhang, Fan Tong, Ruiguang Zhang, Zhaoyi Liu, Lin Zhang, Juan Yu, Xue Yang, Yang Xia, Xiaorong Dong, Gao Zhang, Nong Yang, and Yongchang Zhang

Characteristics	Total(N=226)	HR(95% CI) Subgroup analysis	P value
Age			
≥55	125	0.45（0.31-0.66）	<0.001
<55	101	0.57（0.38-0.86）	0.007
Sex			
Male	102	0.45（0.29-0.70）	<0.001
Female	124	0.54（0.37-0.78）	0.001
Smoking history			
Never smoker	142	0.55（0.39-0.78）	0.001
Smoker	84	0.40（0.25-0.66）	<0.001
PS score			
Low(0-1)	222	0.50（0.37-0.66）	<0.001
High(2-3)	4	-	-
Liver metastasis			
With	38	0.56（0.29-1.11）	0.09
Without	188	0.48（0.35-0.65）	<0.001
Brain metastasis			
With	43	0.27（0.13-0.57）	0.001
Without	183	0.54(0.40-0.74)	<0.001
EGFR molecular subtype			
19del	128	0.59（0.41-0.85）	0.005
21L858R	72	0.43（0.26-0.70）	0.001
Other mutation	26	0.33（0.13-0.84）	0.02
TP53 concomitant mutation			
With	47	0.69（0.38-1.26）	0.232
Without	179	0.45（0.33-0.62）	<0.001
Treatment line			
Second-line	100	0.50（0.33-0.76）	0.001
Third-line	126	0.48（0.33-0.70）	<0.001

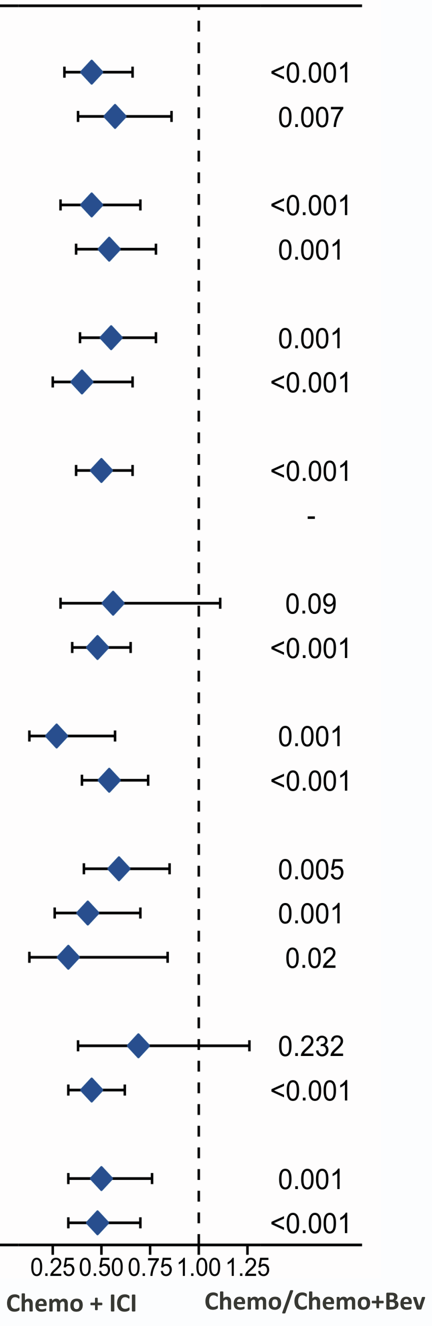

Figure S1. Forest plot illustrating the subgroup analysis of the impact of baseline clinical and molecular factors on progression-free survival (PFS).

Characteristics	Total (N=117)	HR(95% CI) Univariate analysis	P value
Age			
≥55	67	Reference	
<55	50	1.31 (0.89 - 1.91)	0.166
Sex			
Male	53	Reference	
Female	64	1.03 (0.71 - 1.49)	0.872
Smoking history			
Never smoker	71	Reference	
Smoker	46	0.91 (0.62 - 1.34)	0.642
PS score			
Low(0-1)	113	Reference	
High(2-3)	4	1.18 (0.43 - 3.23)	0.745
Liver metastasis			
Without	96	Reference	
With	21	1.20 (0.75 - 1.94)	0.45
Brain metastasis			
Without	96	Reference	
With	21	0.84 (0.52 - 1.36)	0.48
EGFR molecular subtype			
19del	65	Reference	
21L858R	41	0.91 (0.61 - 1.36)	0.649
Other mutation	11	0.62 (0.31 - 1.22)	0.165
TP53 concomitant mutation			
Without	90	Reference	
With	27	1.58 (1.02 - 2.47)	0.042
Treatment line			
Second-line	46	Reference	
Third-line	71	1.04 (0.71 - 1.52)	0.848
PD-L1			
<1%	28	Reference	
1-49%	36	0.92 (0.55 - 1.52)	0.732
≥50%	23	0.88 (0.50 - 1.56)	0.67
Unknow	30	0.85 (0.50 - 1.45)	0.551
TMB			
<10	61	Reference	
≥10	22	0.98 (0.59 - 1.62)	0.94
Unknown	34	1.18 (0.77 - 1.82)	0.438
Immune checkpoint inhibitors			
Pembrolizumab	53	Reference	
Toripalimab	36	1.02 (0.66 - 1.56)	0.946
Sintilimab	28	1.081(0.67 - 1.73)	0.747

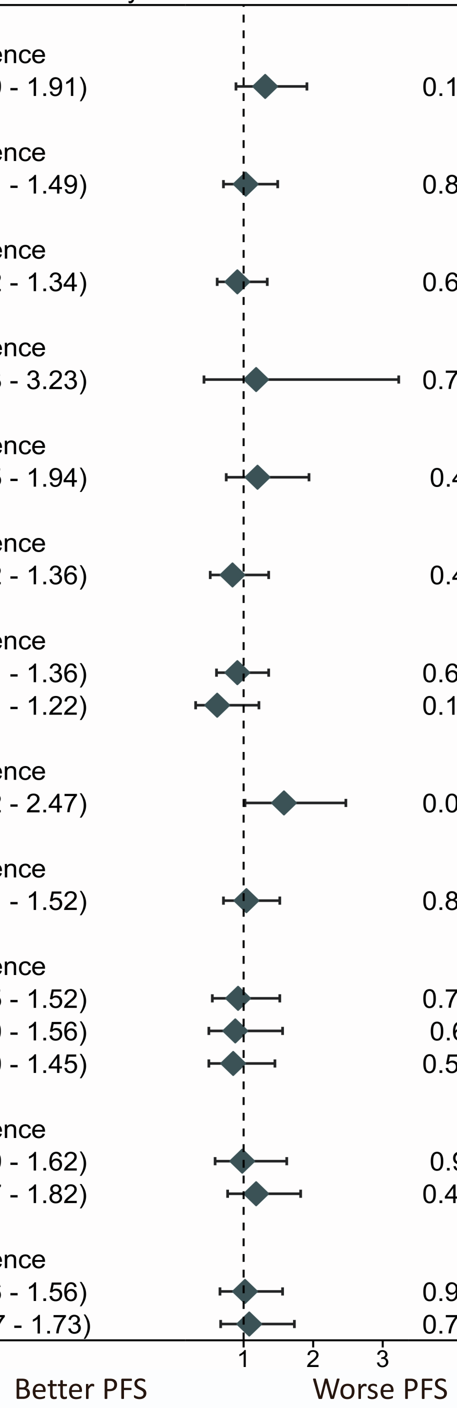

Figure S2. Forest plot summarizing the subgroup analysis for clinicopathological variables associated with progression-free survival (PFS) among patients who received chemotherapy and immune checkpoint inhibitor therapy.

Characteristics	Total(N=226)	HR(95% CI) Univariate analysis	P value	HR(95% CI) Multivariate analysis	P value
Age					
<55	101	Reference		Reference	
≥55	125	0.79 (0.60-1.03)	0.077	0.82 (0.62-1.10)	0.183
Sex					
Female	124	Reference		Reference	
Male	102	1.04 (0.80-1.36)	0.769	1.24 (0.77-1.99)	0.378
Smoking history					
Never smoker	142	Reference		Reference	
Smoker	84	0.96 (0.73-1.26)	0.754	0.96 (0.59-1.56)	0.869
PS score					
High(2-3)	4	Reference		Reference	
Low(0-1)	222	1.18 (0.44-3.18)	0.742	1.14 (0.39-3.32)	0.817
Liver metastasis					
With	38	Reference		Reference	
Without	188	0.94 (0.66-1.33)	0.722	0.90 (0.63-1.30)	0.58
Brain metastasis					
With	43	Reference		Reference	
Without	183	1.01 (0.72-1.42)	0.945	0.93 (0.66-1.31)	0.675
EGFR molecular subtype					
19del	128	Reference		Reference	
21L858R	72	0.96 (0.72-1.29)	0.797	1.12 (0.82-1.52)	0.479
Other mutation	26	0.95 (0.61-1.47)	0.814	1.03 (0.66-1.62)	0.881
TP53 concomitant mutation					
With	47	Reference		Reference	
Without	179	0.77 (0.55-1.06)	0.11	0.72 (0.51-1.03)	0.071
Treatment reagent					
Chemo	72	Reference		Reference	
Chemo+Bev	37	0.76 (0.51-1.14)	0.183	0.67 (0.44-1.02)	0.061
Chemo+ICI	117	0.52 (0.37-0.73)	<0.001	0.41 (0.29-0.57)	<0.001
Treatment line					
Second-line	100	Reference		Reference	
Third-line	126	1.03 (0.79-1.34)	0.84	1.19 (0.90-1.56)	0.225

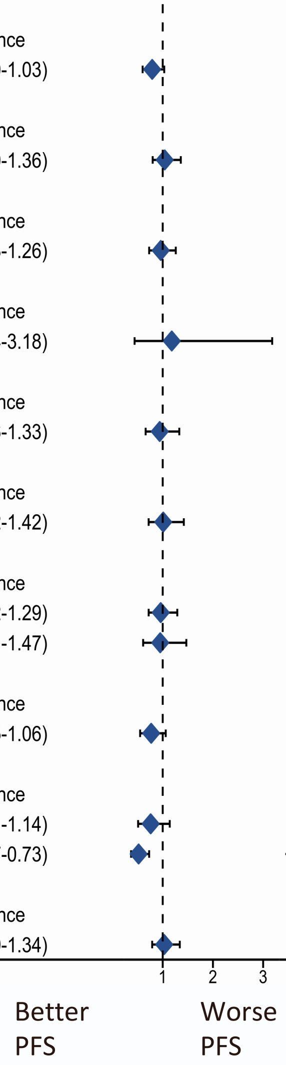

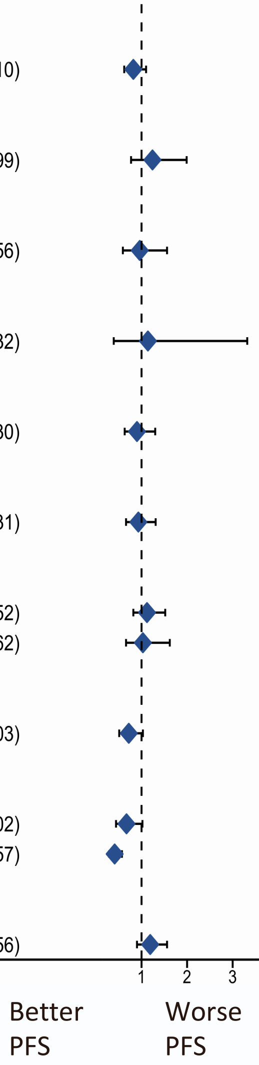

Figure S3. Forest plot summarizing the univariate and multivariate Cox regression analyses for progression-free survival (PFS).

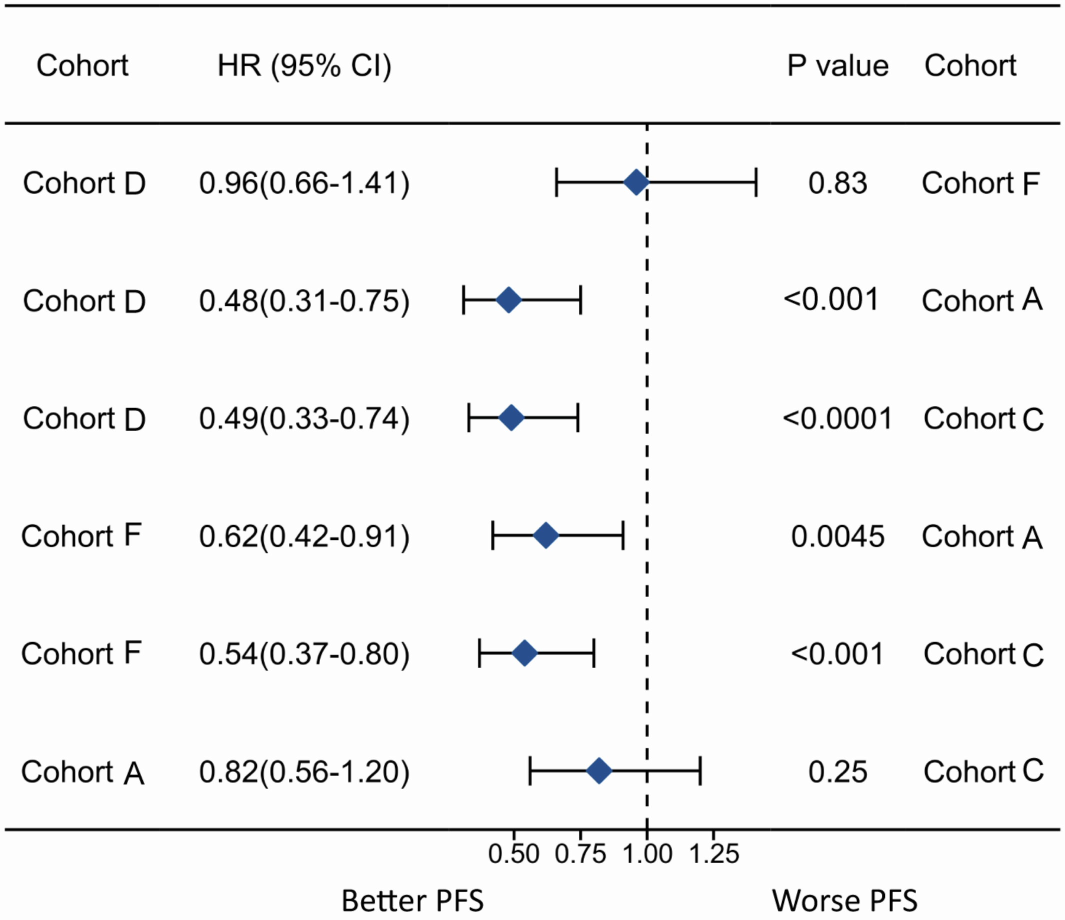

Figure S4. Forest plot summarizing the hazard ratio (HR) and P value for comparing the progression-free survival (PFS) between different cohorts based on treatment history as summarized in the flow diagram in Figure 1. Cohort A refers to patients who progressed from first-line first-/second-generation EGFR-TKI and received Chemo/Chemo+Bev as second-line therapy (n=51). Cohort B refers to patients who progressed from first-line third-generation EGFR-TKI and received Chemo/Chemo+Bev as second-line therapy (n=3). Cohort C refers to patients who progressed from second-line third-generation EGFR-TKI and received Chemo/Chemo+Bev as third-line therapy (n=55).Cohort D refers to patients who progressed from first-line first-/second-generation EGFR-TKI and received Chemo+ICIs as second-line therapy (n=44). Cohort E refers to patients who progressed from first-line third-generation EGFR-TKI and received Chemo+ICIs as second-line therapy (n=2). Cohort F refers to patients who progressed from second-line third-generation EGFR-TKI and received Chemo+ICIs as third-line therapy (n=71).

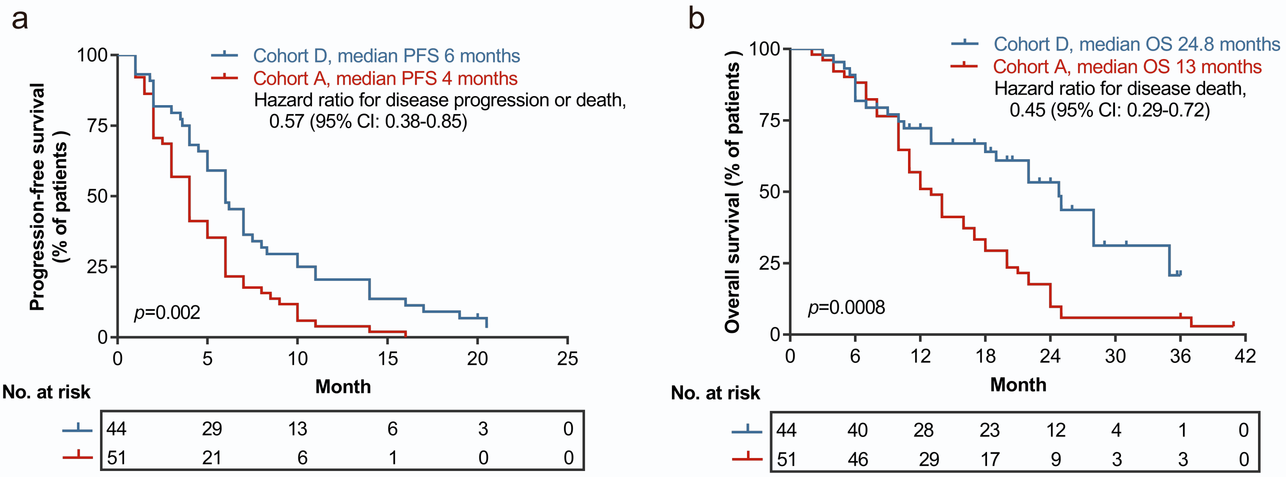

Figure S5. Survival analysis based on EGFR-TKI treatment history. Kaplan-Meier curves comparing the progression-free survival (PFS) (**a**) and overall survival (OS) (**b**) between Cohorts A and D. Cohort A refers to patients whose disease progressed on first-line first-/second-generation EGFR-TKI and received Chemo/Chemo+Bev as second-line therapy (n=51). Cohort D represents patients whose disease progressed on first-line first-/second-generation EGFR-TKI and received Chemo+ICIs as second-line therapy (n=44). Tick marks indicate censored data. Risk table below summarizes the number of patients analyzed per time point. Abbreviation: CI, confidence intervals.

Characteristics	Total(N=226)	HR(95% CI) Subgroup analysis	P value
Age			
≥55	125	0.51(0.34-0.78)	0.002
<55	101	0.74(0.47-1.16)	0.192
Sex			
Male	102	0.52(0.33-0.82)	0.005
Female	124	0.65(0.44-0.96)	0.031
Smoking history			
Never smoker	142	0.63(0.44-0.92)	0.016
Smoker	84	0.51(0.31-0.84)	0.008
PS score			
Low(0-1)	222	0.59(0.44-0.80)	0.001
High(2-3)	4	-	-
Liver metastasis			
With	38	0.42(0.20-0.88)	0.021
Without	188	0.64(0.46-0.89)	0.007
Brain metastasis			
With	43	0.39(0.19-0.81)	0.011
Without	183	0.65(0.47-0.90)	0.009
EGFR molecular subtype			
19del	128	0.60(0.40-0.88)	0.009
21L858R	72	0.89(0.53-1.52)	0.676
Other mutation	26	0.10(0.03-0.38)	0.001
TP53 concomitant mutation			
With	47	0.67(0.35-1.31)	0.242
Without	179	0.58(0.42-0.81)	0.002
Treatment line			
Second-line	100	0.45(0.28-0.73)	0.001
Third-line	126	0.71(0.48-1.04)	0.08

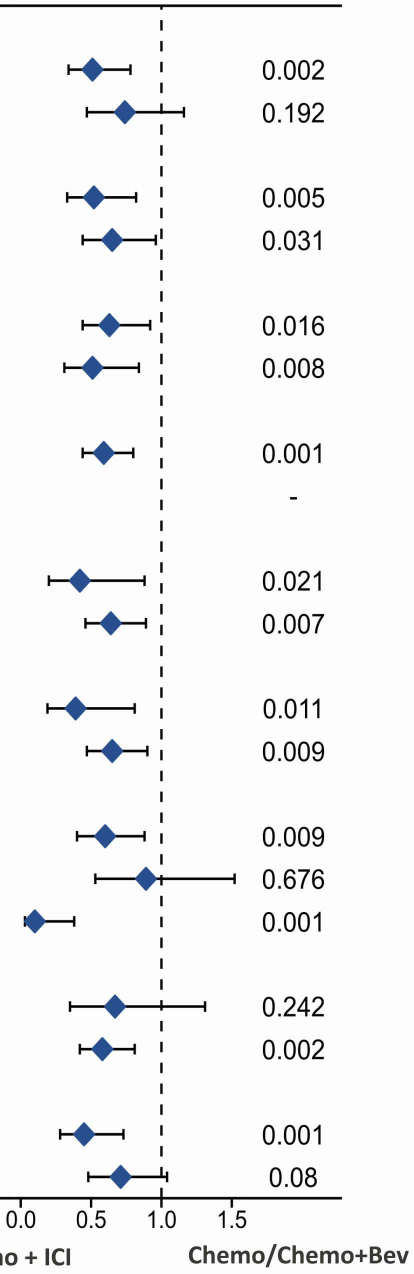

Figure S6. Forest plot illustrating the subgroup analysis on the impact of baseline clinical and molecular factors on overall survival (OS) with Chemo+ICI and Chemo/Chemo+Bev.

Characteristics	Total(N=226)	HR(95% CI) Univariate analysis	P value	HR(95% CI) Multivariate analysis	P value
Age					
<55	101	Reference		Reference	
≥55	125	0.86 (0.64-1.15)	0.302	0.93 (0.69-1.27)	0.668
Sex					
Female	124	Reference		Reference	
Male	102	0.98 (0.73-1.31)	0.883	0.89 (0.52-1.53)	0.674
Smoking history					
Never smoker	142	Reference		Reference	
Smoker	84	0.92 (0.68-1.25)	0.609	1.09 (0.62-1.90)	0.773
PS score					
High(2-3)	4	Reference		Reference	
Low(0-1)	222	0.76 (0.28-2.05)	0.582	0.67 (0.21-2.11)	0.495
Liver metastasis					
With	38	Reference		Reference	
Without	188	1.28 (0.86-1.90)	0.233	1.29 (0.86-1.95)	0.222
Brain metastasis					
With	43	Reference		Reference	
Without	183	0.73 (0.50-1.06)	0.096	0.72 (0.49-1.06)	0.094
EGFR molecular subtype					
19del	128	Reference		Reference	
21L858R	72	0.81 (0.59-1.13)	0.223	0.82 (0.58-1.17)	0.275
Other mutation	26	1.13 (0.70-1.83)	0.611	1.25 (0.76-2.05)	0.388
TP53 concomitant mutation					
With	47	Reference		Reference	
Without	179	0.87 (0.60-1.26)	0.464	0.90 (0.61-1.33)	0.612
Treatment reagent					
Chemo	72	Reference		Reference	
Chemo+Bev	37	1.13 (0.75-1.69)	0.568	1.08 (0.71-1.65)	0.708
Chemo+ICI	117	0.64 (0.46-0.91)	0.006	0.59 (0.42-0.84)	0.003
Treatment line					
Second-line	100	Reference		Reference	
Third-line	126	1.19 (0.88-1.60)	0.256	1.24 (0.91-1.68)	0.168

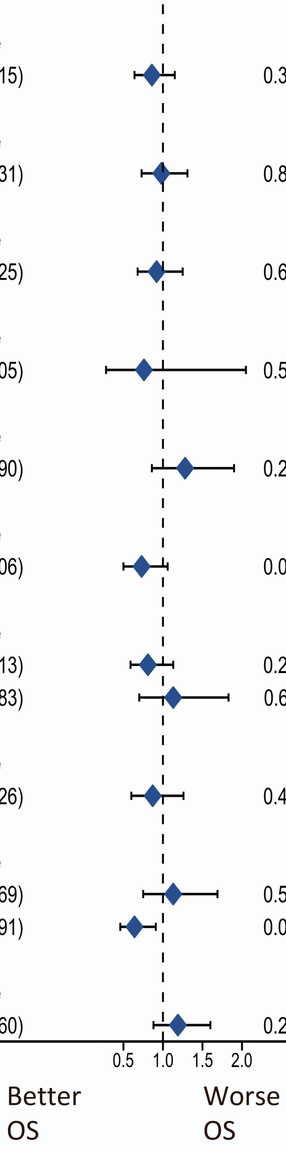

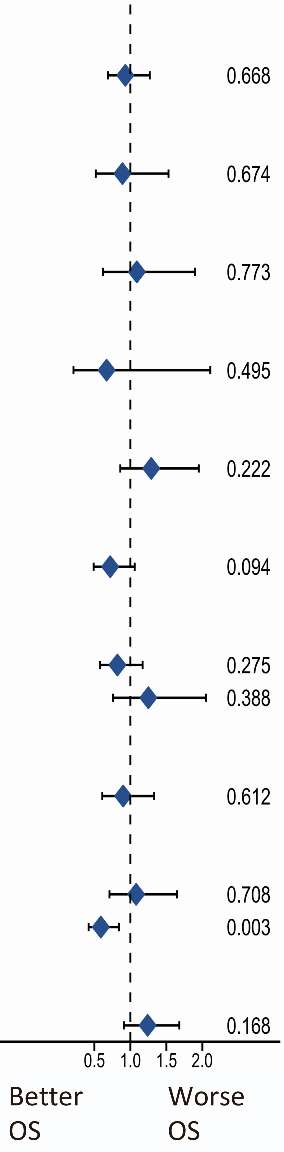

Figure S7. Forest plot summarizing the univariate and multivariate Cox regression analyses on the impact of various baseline clinical and molecular factors on overall survival (OS).

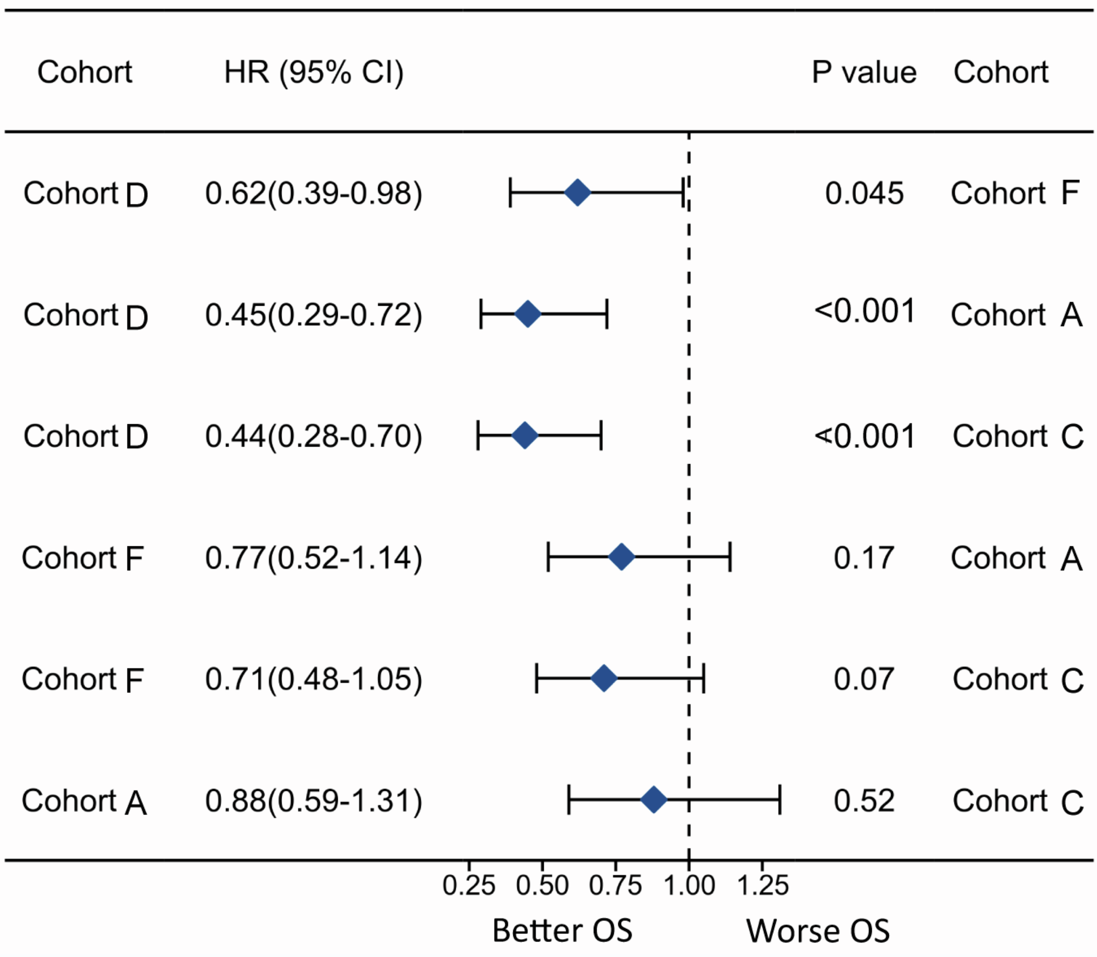

Figure S8. Forest plot summarizing the hazard ratio (HR) and P value for comparing the overall survival (OS) between different cohorts based on treatment history as summarized in the flow diagram in Figure 1. Cohort A refers to patients who progressed from first-line first-/second-generation EGFR-TKI and received Chemo/Chemo+Bev as second-line therapy (n=51). Cohort B refers to patients who progressed from first-line third-generation EGFR-TKI and received Chemo/Chemo+Bev as second-line therapy (n=3). Cohort C refers to patients who progressed from second-line third-generation EGFR-TKI and received Chemo/Chemo+Bev as third-line therapy (n=55). Cohort D refers to patients who progressed from first-line first-/second-generation EGFR-TKI and received Chemo+ICIs as second-line therapy (n=44). Cohort E refers to patients who progressed from first-line third-generation EGFR-TKI and received Chemo+ICIs as second-line therapy (n=2). Cohort F refers to patients who progressed from second-line third-generation EGFR-TKI and received Chemo+ICIs as third-line therapy (n=71).

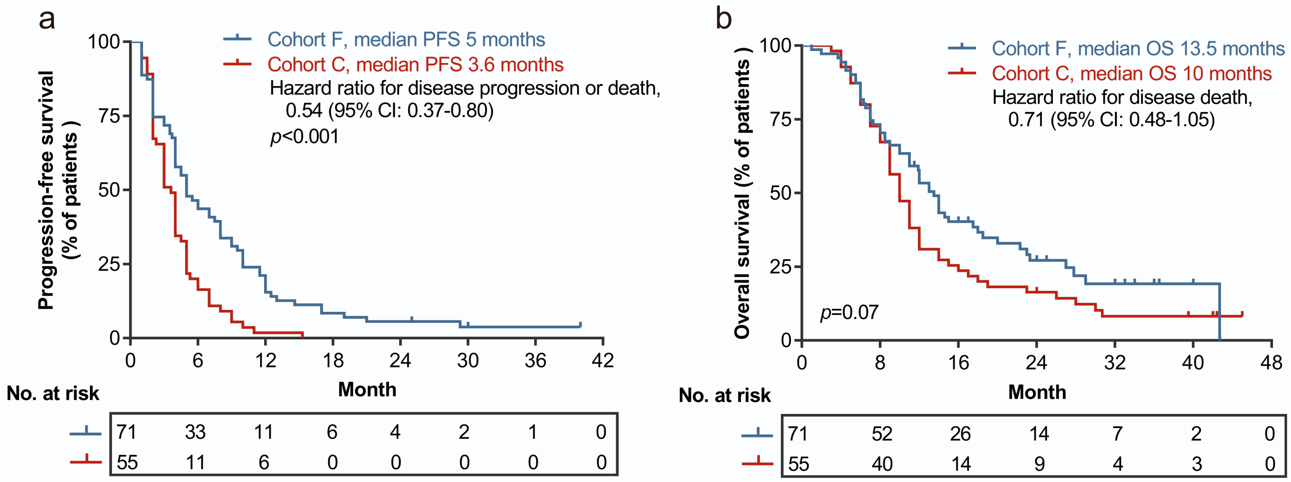

Figure S9. Survival analysis based on EGFR-TKI treatment history. Kaplan-Meier curves comparing the progression-free survival (PFS) (**a**) and overall survival (OS) (**b**) between Cohorts C and F. Cohort C represented patients who progressed on second-line third-generation EGFR-TKI and received Chemo/Chemo+Bev as third-line therapy (n=55). Cohort F represents patients who progressed on second-line third-generation EGFR-TKI and received Chemo+ICIs as third-line therapy (n=71). Tick marks indicate censored data. Risk table below summarizes the number of patients analyzed per time point. Abbreviation: CI, confidence intervals.

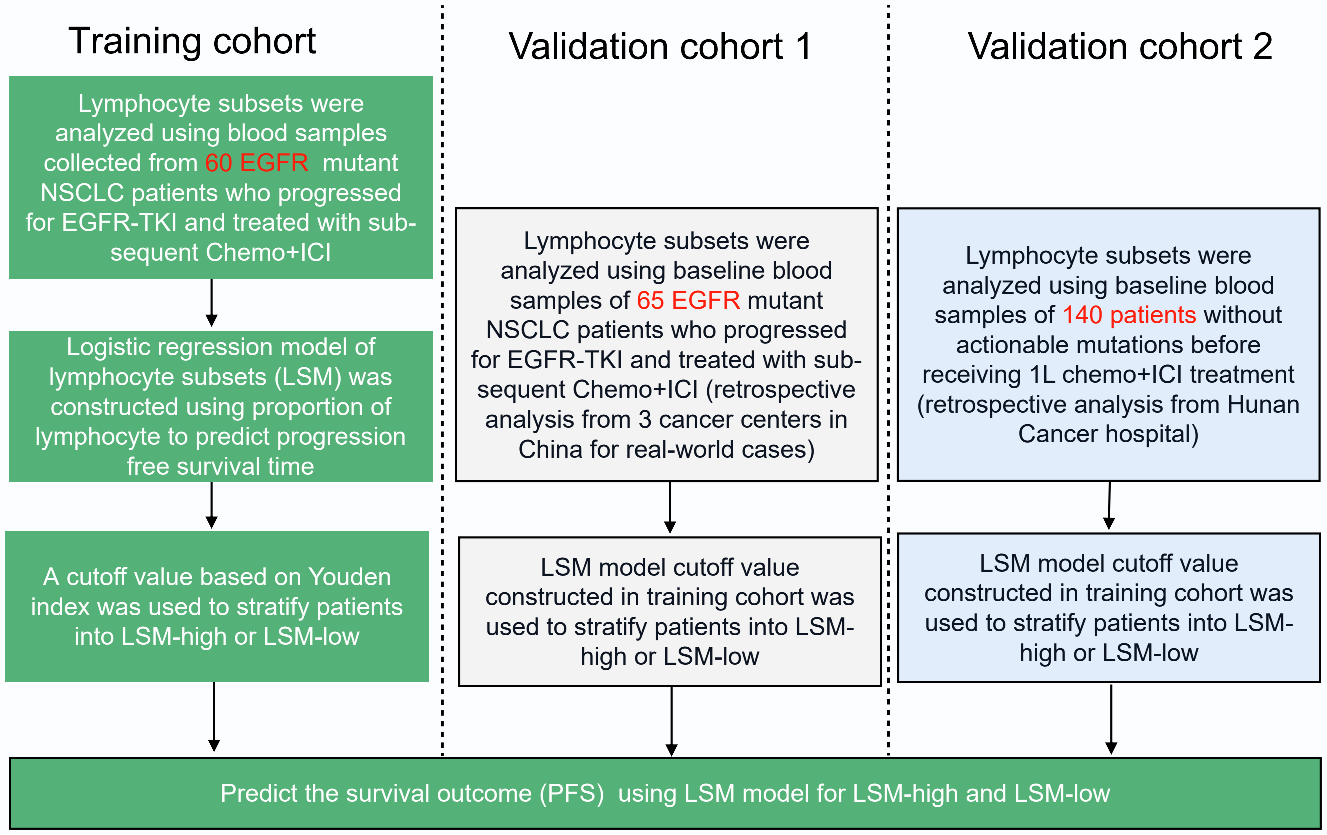

Figure S10. Schematic diagram showing the study design and cohorts used for the training and validation of the lymphocyte subsets model.

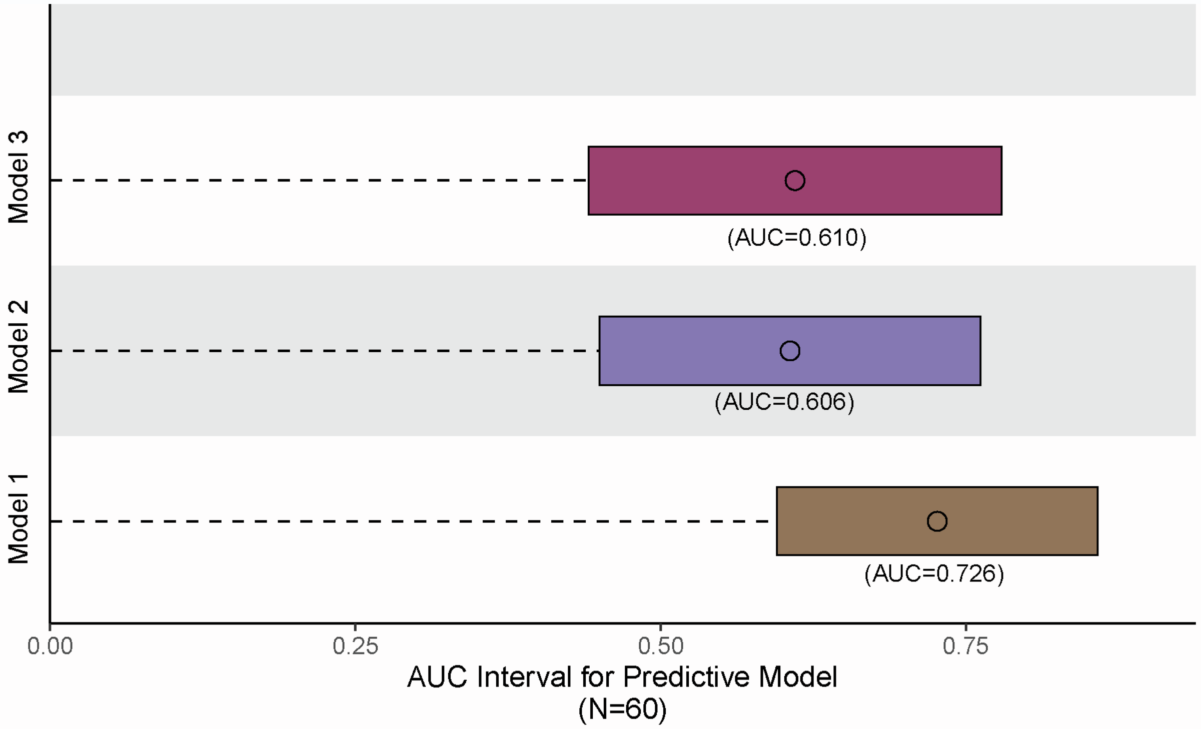

Figure S11. Selection of the optimal logistic regression-based lymphocyte subset model (LSM) for predicting the clinical outcomes of Chemo+ICI in patients with NSCLC. The plot shows the area under the curve (AUC) for three models that were initially constructed using the treatment outcome and lymphocyte subset data from 60 patients in the training cohort to predict progression-free survival (PFS) of ≥9.0 months for Model 1, PR for Model 2, and having disease progression at data cutoff date for Model 3.

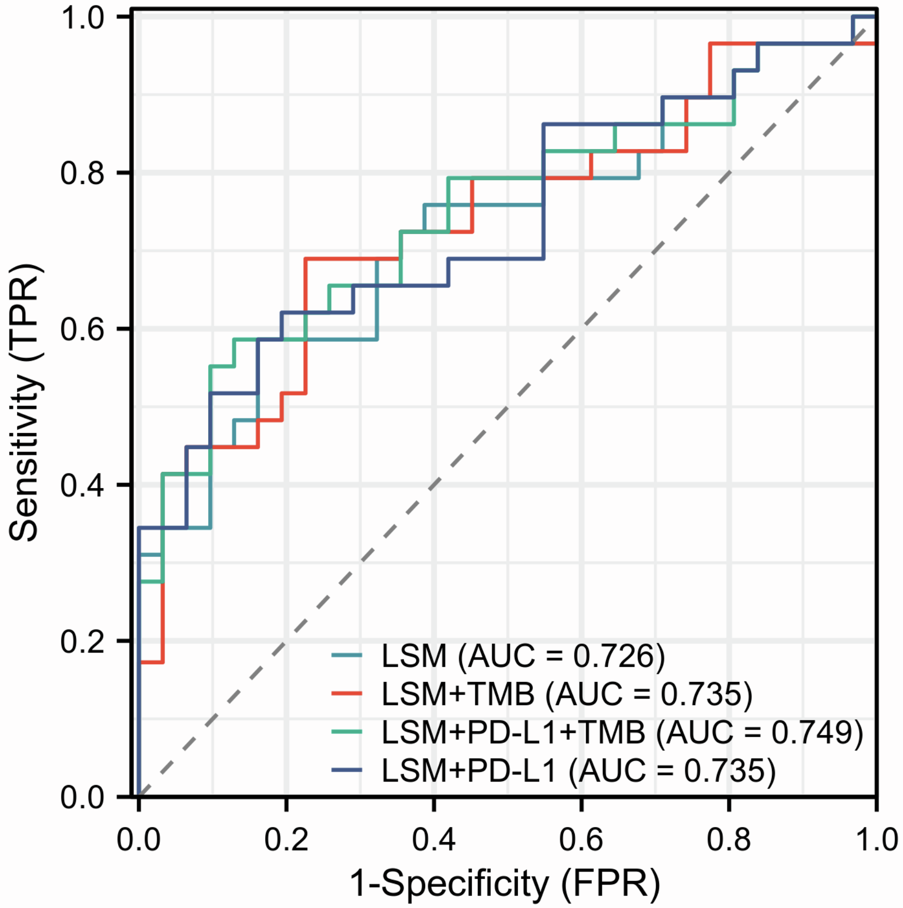

Variable	AUC	95% CI
LSM+PD-L1+TMB	0.749	0.621 to 0.877
LSM+PD-L1	0.735	0.605 to 0.865
LSM+TMB	0.735	0.605 to 0.865
LSM	0.726	0.595 to 0.858

Figure S12. Receiver operating characteristics curve showing the area under the curve (AUC) for predictive performance with LSM combined with PD-L1 expression and tumor mutation burden (TMB) analysis.

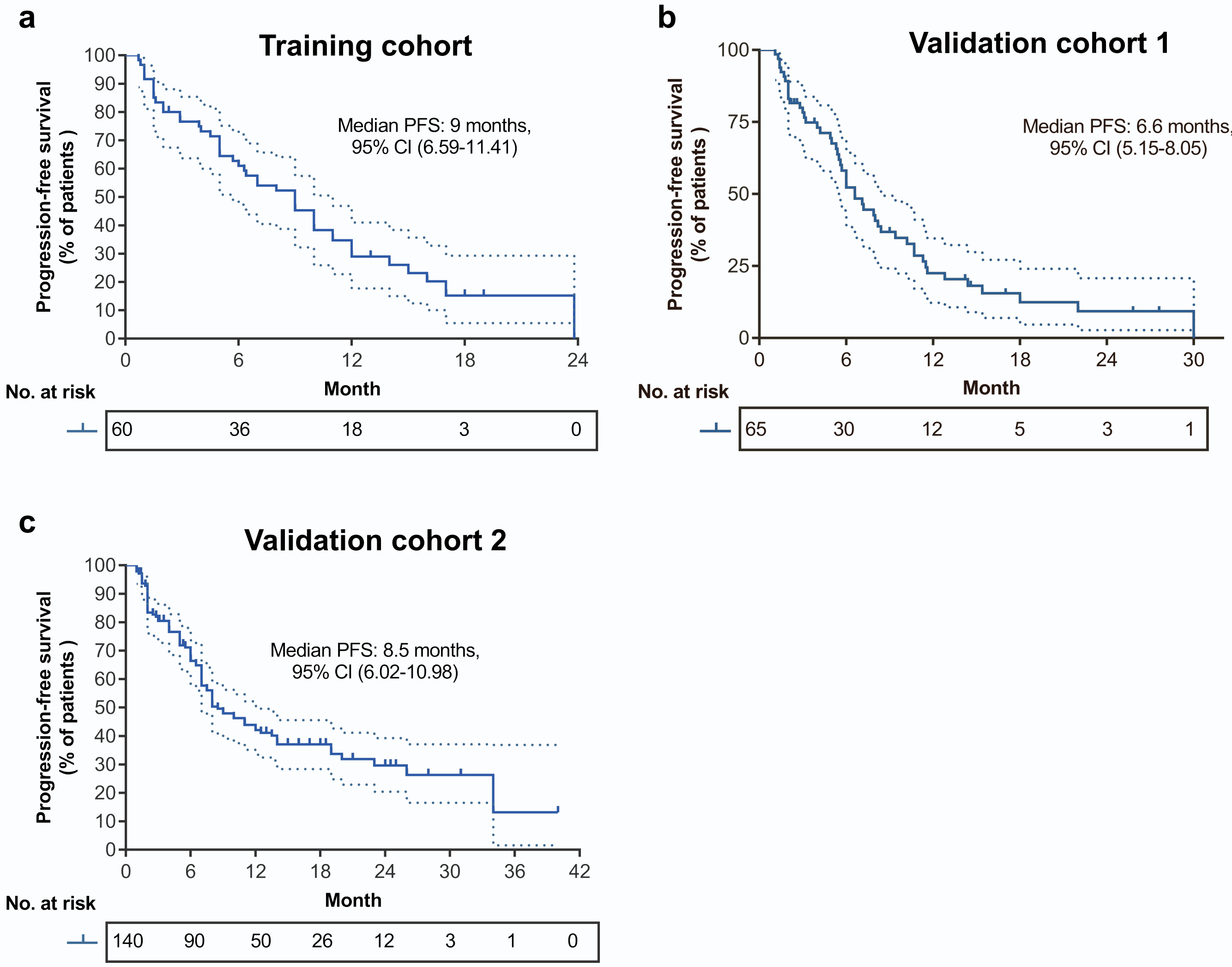

Figure S13. Progression-free survival of training and validation cohorts. Kaplan-Meier survival curves showing the progression-free survival (PFS) for the training cohort (**a**), validation cohort 1 (**b**), and validation cohort 2 (**c**). Tick marks indicate censored data. Risk table below summarizes the number of patients analyzed per time point. Abbreviation: CI, confidence intervals.

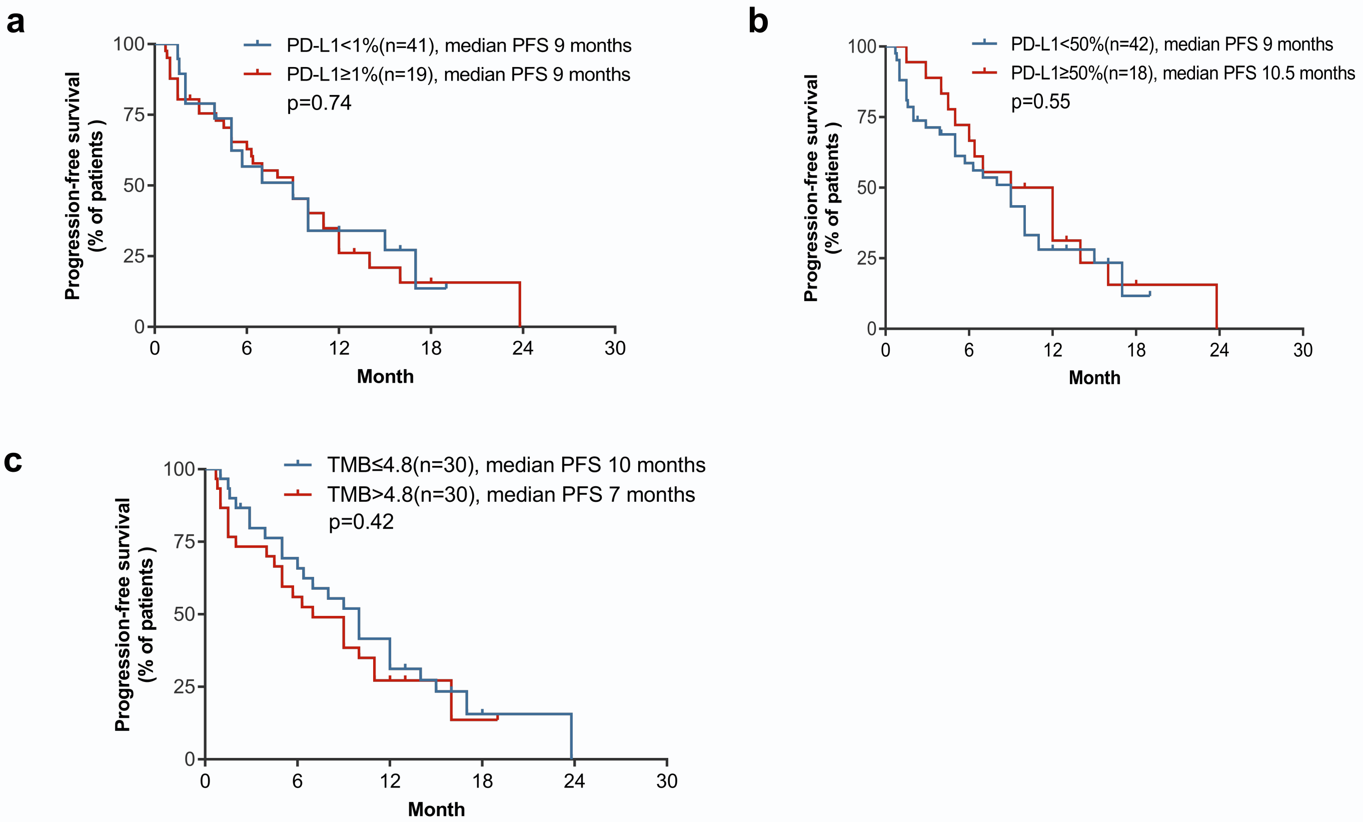

Figure S14. **Validation of predictive model based on PD-L1 expression and tumor mutation burden (TMB)**. Kaplan-Meier survival curves comparing the progression-free survival (PFS) between patients in the training cohort with PD-L1 tumor proportion score <1% and ≥1% (**a**) or <50% and ≥50% (**b**) and TMB > 4.8 and ≤ 4.8 mutations/Mb. Tick marks indicate censored data. Risk table below summarizes the number of patients analyzed per time point. Abbreviation: CI, confidence intervals.

Characteristics	Total (N=60)	HR(95% CI) Univariate analysis	P value	HR(95% CI) Multivariate analysis	P value
Sex					
Male	21	Reference		Reference	
Female	39	1.25 (0.67 - 2.33)	0.476	1.13 (0.52 - 2.46)	0.766
Age	60	0.97 (0.95 - 1.01)	0.114	0.98 (0.95 - 1.02)	0.287
Smoking history					
Never	45	Reference		Reference	
Former	15	0.73 (0.36 - 1.49)	0.396	0.65 (0.26 - 1.59)	0.349
PD-L1					
<1%	19	Reference		Reference	
1-49%	23	1.31 (0.63 - 2.69)	0.459	1.03 (0.45 - 2.32)	0.953
≥50%	18	0.94 (0.45 - 1.99)	0.887	0.64 (0.27 - 1.49)	0.304
TMB					
0-5	34	Reference		Reference	
6-10	14	1.05 (0.51 - 2.20)	0.886	0.75 (0.33 - 1.70)	0.487
>10	12	1.28 (0.60 - 2.76)	0.517	0.90 (0.38 - 2.15)	0.818
LSM score	60	0.72 (0.54 - 0.96)	0.024	0.67 (0.47 - 0.98)	0.038

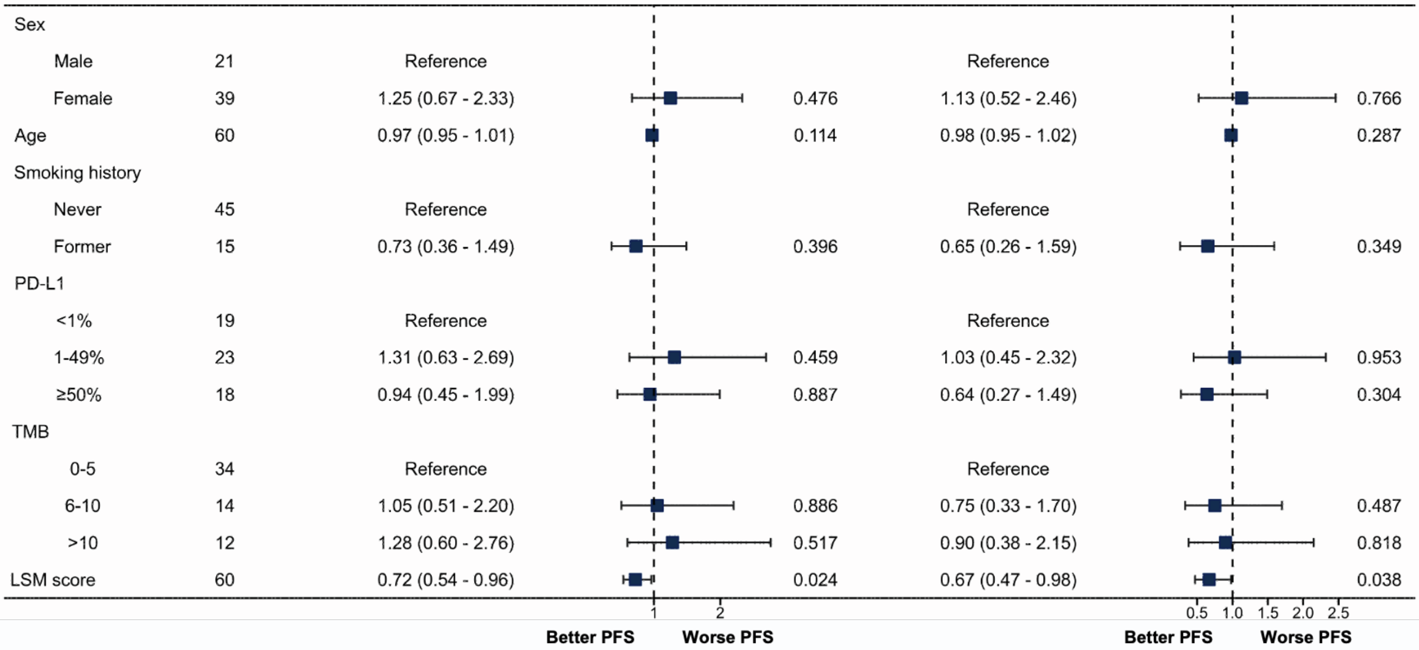

Figure S15. Forest plot summarizing the univariate and multivariate Cox regression analyses for progression-free survival (PFS) in the training cohort. Abbreviations: CI, confidence intervals; HR, hazard ratio; TMB, tumor mutation burden.

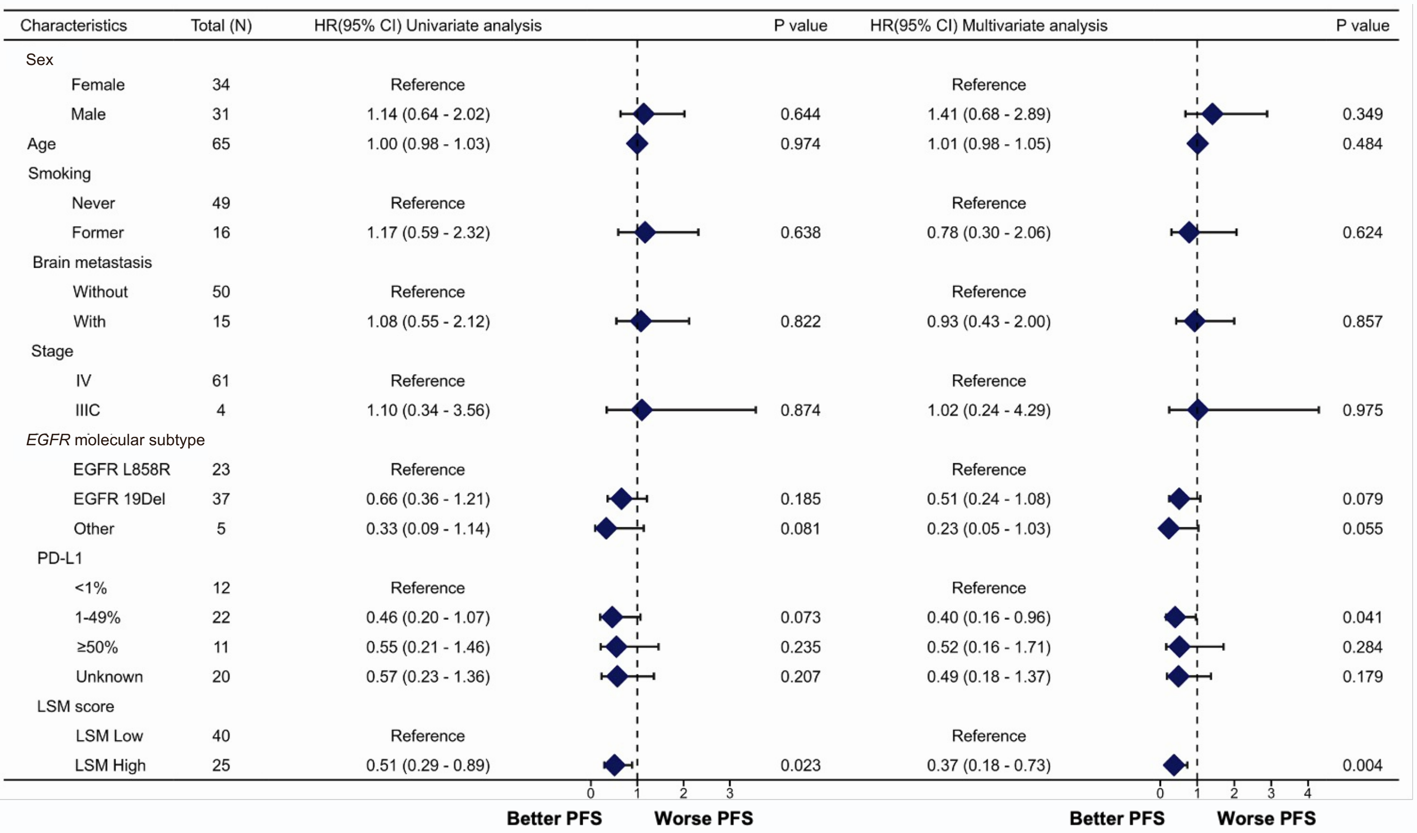

Characteristics	Total (N)	HR(95% CI) Univariate analysis	P value	HR(95% CI) Multivariate analysis	P value
Sex					
Female	34	Reference		Reference	
Male	31	1.14 (0.64 - 2.02)	0.644	1.41 (0.68 - 2.89)	0.349
Age	65	1.00 (0.98 - 1.03)	0.974	1.01 (0.98 - 1.05)	0.484
Smoking					
Never	49	Reference		Reference	
Former	16	1.17 (0.59 - 2.32)	0.638	0.78 (0.30 - 2.06)	0.624
Brain metastasis					
Without	50	Reference		Reference	
With	15	1.08 (0.55 - 2.12)	0.822	0.93 (0.43 - 2.00)	0.857
Stage					
IV	61	Reference		Reference	
IIIC	4	1.10 (0.34 - 3.56)	0.874	1.02 (0.24 - 4.29)	0.975
EGFR molecular subtype					
EGFR L858R	23	Reference		Reference	
EGFR 19Del	37	0.66 (0.36 - 1.21)	0.185	0.51 (0.24 - 1.08)	0.079
Other	5	0.33 (0.09 - 1.14)	0.081	0.23 (0.05 - 1.03)	0.055
PD-L1					
<1%	12	Reference		Reference	
1-49%	22	0.46 (0.20 - 1.07)	0.073	0.40 (0.16 - 0.96)	0.041
≥50%	11	0.55 (0.21 - 1.46)	0.235	0.52 (0.16 - 1.71)	0.284
Unknown	20	0.57 (0.23 - 1.36)	0.207	0.49 (0.18 - 1.37)	0.179
LSM score					
LSM Low	40	Reference		Reference	
LSM High	25	0.51 (0.29 - 0.89)	0.023	0.37 (0.18 - 0.73)	0.004

Figure S16. Forest plot summarizing the univariate and multivariate Cox regression analyses for progression-free survival (PFS) in the validation cohort 1. Abbreviations: CI, confidence intervals; HR, hazard ratio.

Characteristics	Total (N=140)	HR(95% CI) Univariate analysis	P value	HR(95% CI) Multivariate analysis	P value
Sex					
Male	109	Reference		Reference	
Female	31	0.95 (0.57 - 1.59)	0.865	0.69 (0.32 - 1.48)	0.344
Age	140	1.01 (0.99 - 1.04)	0.208	1.02 (0.99 - 1.05)	0.138
Smoking history					
Never	44	Reference		Reference	
Former	96	0.87 (0.55 - 1.36)	0.553	0.61 (0.31 - 1.18)	0.141
Liver metastases					
Without	124	Reference		Reference	
With	16	1.47 (0.76 - 2.86)	0.248	0.89 (0.44 - 1.82)	0.766
Brain metastases					
Without	117	Reference		Reference	
With	23	0.82 (0.44 - 1.52)	0.539	0.74 (0.38 - 1.43)	0.377
Pathology diagnosis					
ADC	68	Reference		Reference	
SCC	72	1.01 (0.66 - 1.55)	0.942	0.69 (0.42 - 1.13)	0.142
PD-L1 level					
<1%	24	Reference		Reference	
1-49%	25	1.11 (0.51 - 2.36)	0.796	1.02 (0.47 - 2.19)	0.955
≥50%	18	0.67 (0.26 - 1.73)	0.417	0.77 (0.28 - 2.10)	0.614
Unknown	73	1.95 (1.03 - 3.68)	0.04	2.20 (1.13 - 4.29)	0.021
LSM score					
LSM low	83	Reference		Reference	
LSM high	57	0.61 (0.38 - 0.95)	0.031	0.61 (0.38 - 0.97)	0.038

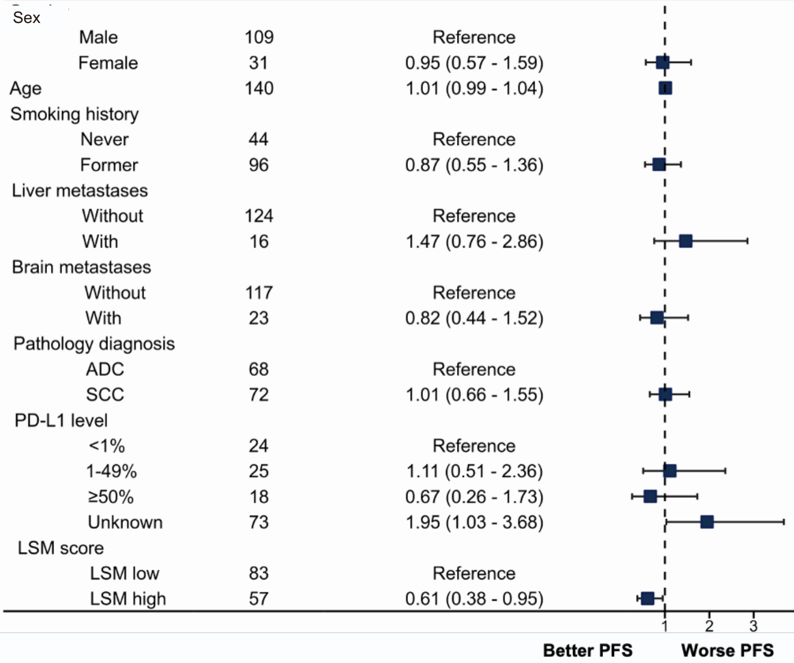

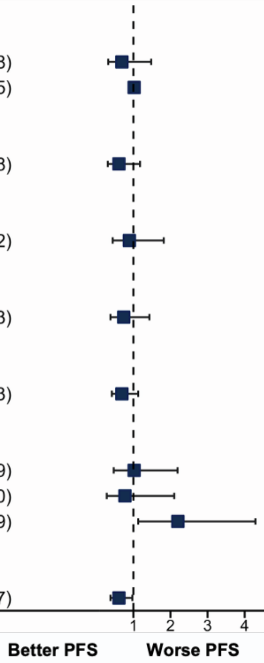

Figure S17. Forest plot summarizing the univariate and multivariate Cox regression analyses for progression-free survival (PFS) in the validation cohort 2. Abbreviations: CI, confidence intervals; HR, hazard ratio.

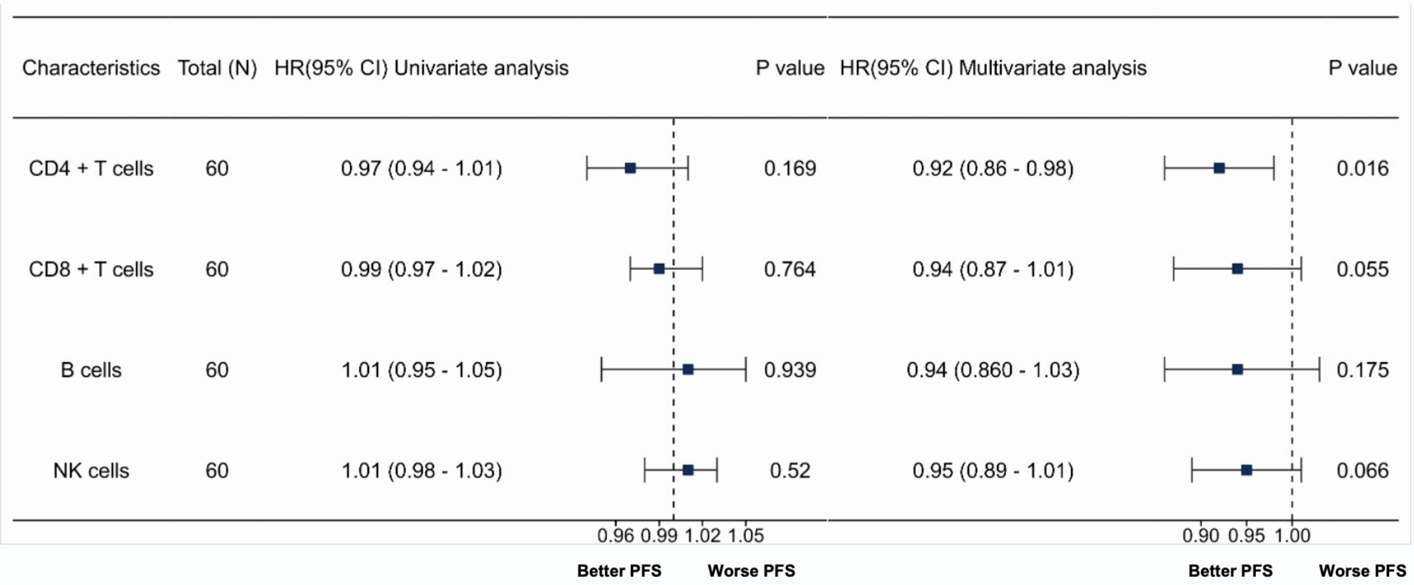

Figure S18. Forest plot summarizing the univariate and multivariate Cox regression analyses for progression-free survival (PFS) when using different subgroups of lymphocyte subsets. Abbreviations: CI, confidence intervals; HR, hazard ratio.

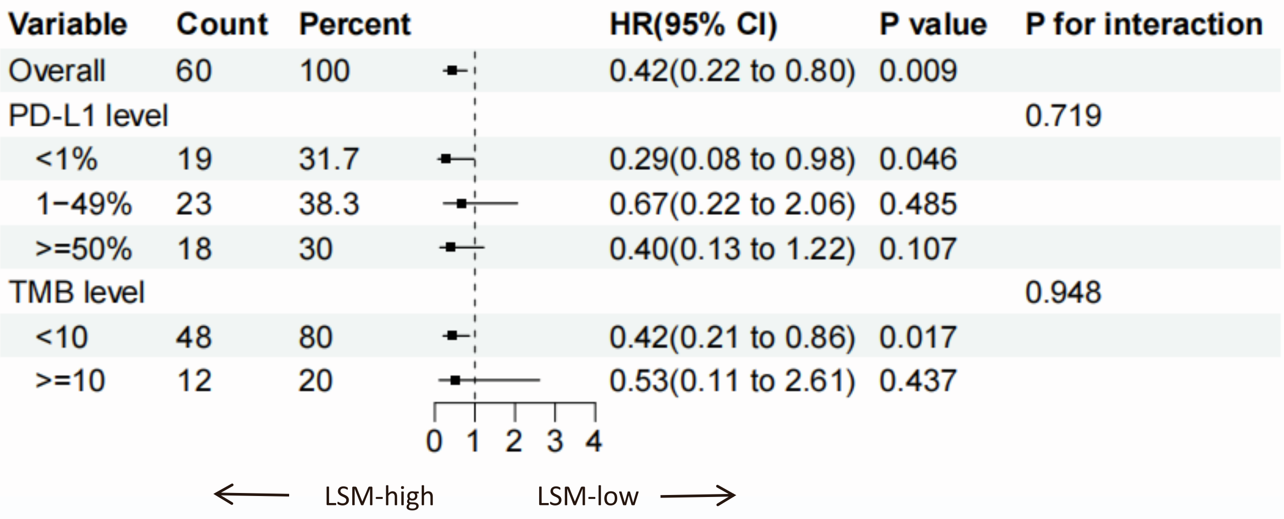

Figure S19.Forest plot showing the hazard ratio (HR) and corresponding 95% confidence intervals (CI) for comparing the impact of tumor mutation burden (TMB) and PD-L1 expression on the progression-free survival (PFS) of LSM-low and LSM-high subgroups of the training cohort.

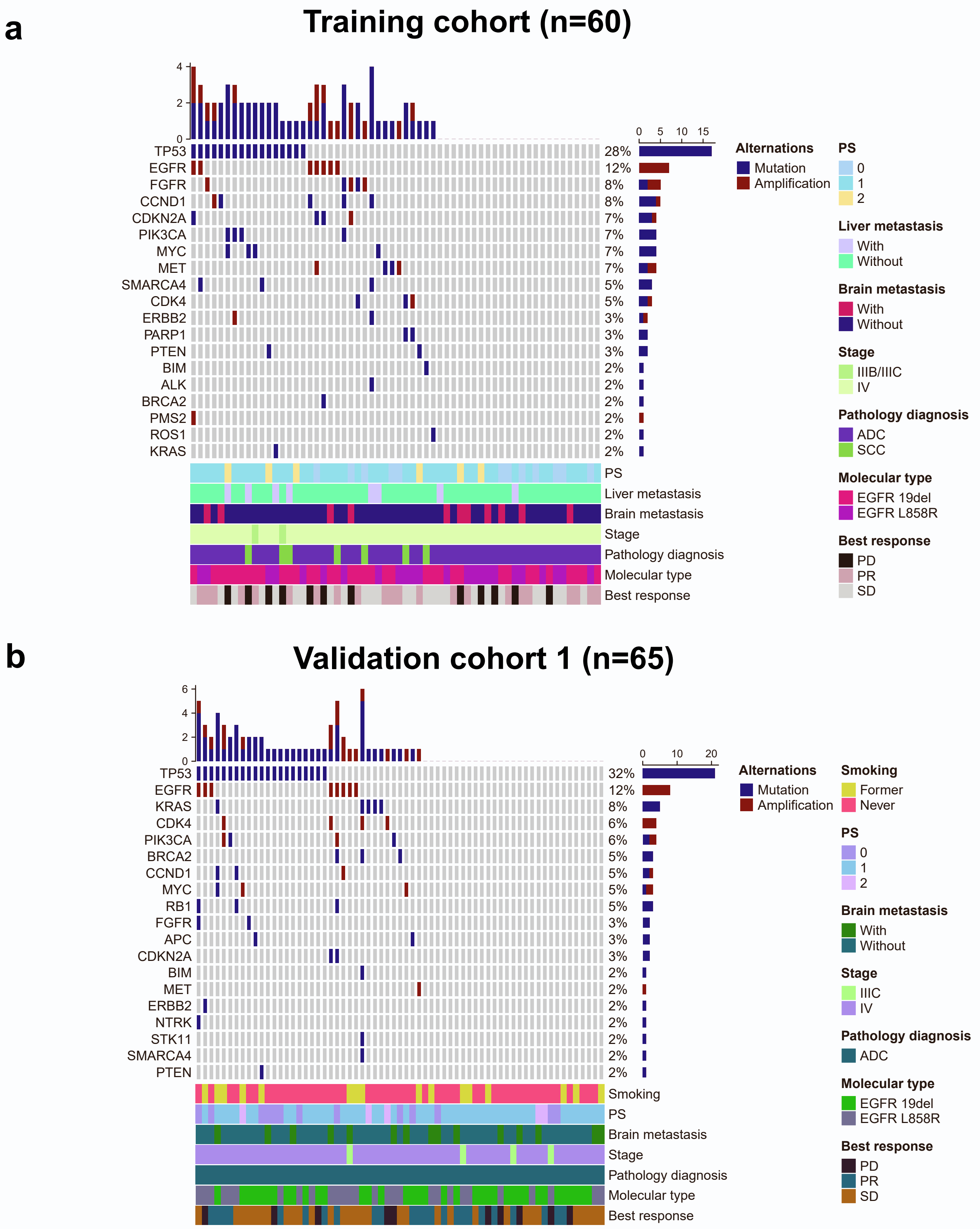

Figure S20. Oncoplot showing the molecular profiles of evaluable patients in the training cohort (**a**) and validation cohort 1 (**b**) at disease progression after first-line EGFR-TKI therapy. The numbers on the left represent the mutation rates of the genes labeled on the right, with different colors indicating various mutation types. The bar graph on the right displays the proportion of each mutation type detected across the genes.

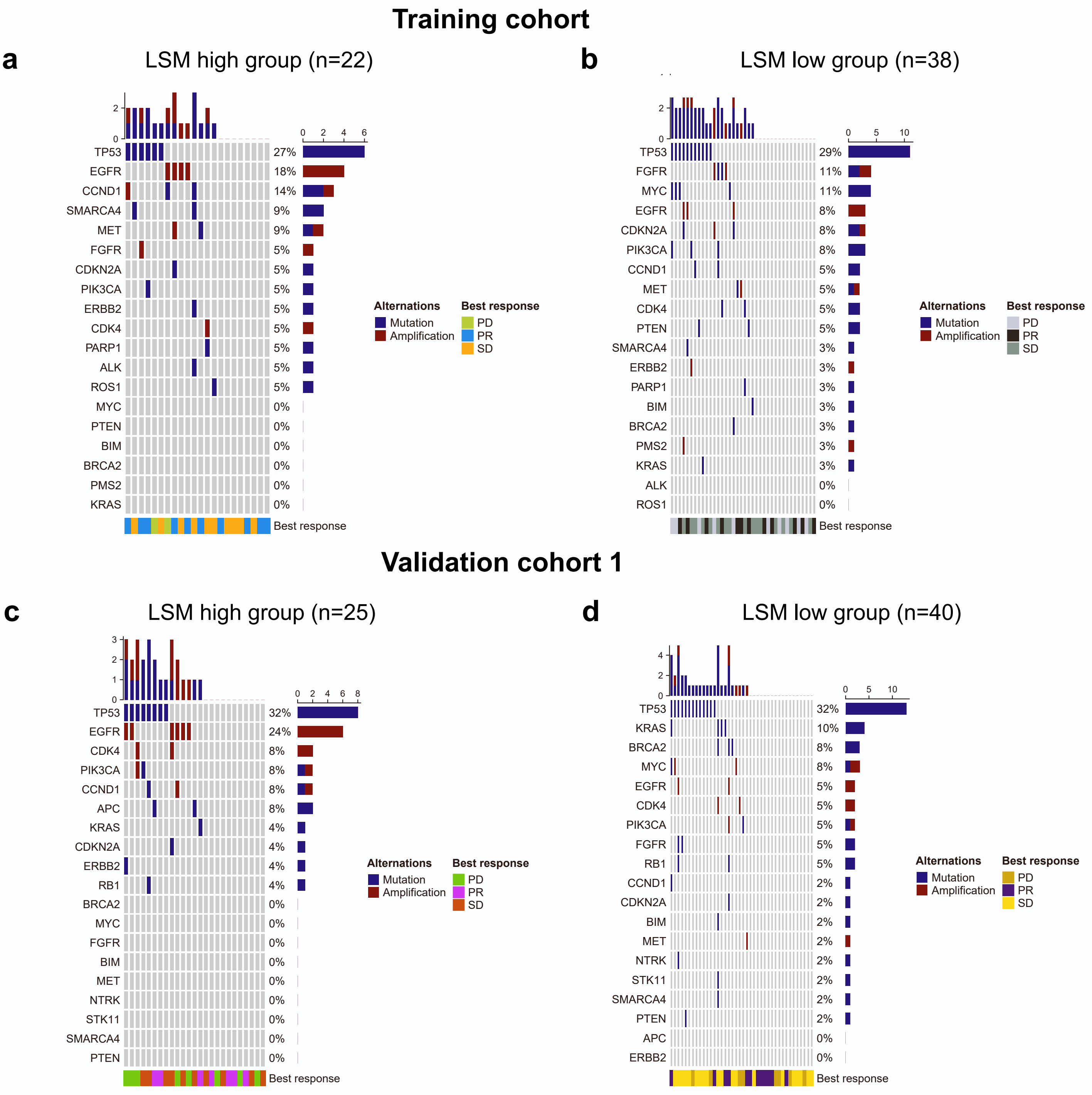

Figure S21. Oncoplot showing the molecular profiles of evaluable patients in the LSM-high (**a**) and LSM-low (**b**) groups within the training cohort as well as the validation cohort1 (**c** and **d**) at the time of disease progression after first-line EGFR-TKI therapy.

Table S1. Baseline characteristics in training and validation cohorts.

Characteristics	Patients		
	Training cohort (n=60)	Validation cohort 1 (n=65)	Validation cohort 2 (n=140)
Age — yr. (Median, Range)	58.1(34-84)	56.7(39-76)	62.6(33-85)
Sex — no. patients (%)			
Female	39(65.0%)	34(52.3%)	31(22.1%)
Male	21(35.0%)	31(47.7%)	109(77.9%)
Smoking history — no. patients (%)			
Former	15(25.0%)	16(24.6%)	96(68.6%)
Never	45(75.0%)	49(75.4%)	44(31.4%)
ECOG performance status — no. patients (%)			
0	12(20.0%)	13(20.0%)	25(17.8%)
1	42(70.0%)	47(72.3%)	109(77.9%)
2	6(10.0%)	5(7.7%)	6(4.3%)
Tumor histology — no. patients (%)			
Adenocarcinoma	53(88.3%)	65(100%)	68(48.6%)
Squamous cell carcinoma	7(11.7%)	0	72(51.4%)
Stage of disease — no. patients (%)			
IIIB/IIIC	2(3.3%)	4(6.2%)	26(18.6%)
IV	58(96.7%)	61(93.8%)	114(81.4%)
Liver metastasis at baseline			
Yes	8(13.3%)	6(9.2%)	16(11.4%)
No	52(86.7%)	60(91.8%)	124(88.6%)
Brain metastasis at baseline			
Yes	11(18.3%)	15(23.1%)	23(16.4%)
No	49(81.7%)	50(76.9%)	117(83.6%)
PD-L1 Level			
<1%	19(31.7%)	12(18.5%)	24(17.1%)
1-49%	23(38.3%)	22(33.8%)	25(17.9%)
⩾50%	18(30.0%)	11(16.9%)	18(12.9%)
Unknown	0	20(30.8%)	73(52.1%)
LSM score			
LSM high	22(36.7%)	25(38.5%)	57(40.7%)
LSM low	38(63.3%)	40(61.5%)	83(59.3%)
Objective response rate			
PR	22(36.7%)	24(36.9%)	68(48.6%)
SD	26(43.3%)	31(47.7%)	49(35.0%)
PD	12(20.0%)	10(15.4%)	23(16.4%)
ORR	36.70%	36.9%	48.60%
DCR	80.00%	84.6%	83.60%

Table S2. Variance inflation factors among lymphocyte sub-populations.

Characteristics	VIF
CD4+ T cells	4.1372
CD8+ T cells	6.5522
B cells	3.0345
NK cells	5.8446
